# On the Inhibitability of Natural Products Isolated from *Tetradium ruticarpum* towards Tyrosine Phosphatase 1B (PTP1B) and α-Glucosidase (3W37): An In Vitro and In Silico Study

**DOI:** 10.3390/molecules26123691

**Published:** 2021-06-17

**Authors:** Dao-Cuong To, Thanh Q. Bui, Nguyen Thi Ai Nhung, Quoc-Toan Tran, Thi-Thuy Do, Manh-Hung Tran, Phan-Phuoc Hien, Truong-Nhan Ngu, Phan-Tu Quy, The-Hung Nguyen, Huu-Tho Nguyen, Tien-Dung Nguyen, Phi-Hung Nguyen

**Affiliations:** 1 Nano Institute (PHENA), Phenikaa University, Yen Nghia, Ha Dong District, Hanoi 12116, Vietnam; cuong.todao@phenikaa-uni.edu.vn; 2 A&A Green Phoenix Group JSC, Phenikaa Research and Technology Institute (PRATI), 167 Hoang Ngan, Cau Giay District, Hanoi 11313, Vietnam; 3 Department of Chemistry, University of Sciences, Hue University, Hue City 530000, Vietnam; thanh.bui@hueuni.edu.vn (T.Q.B.); ntanhung@hueuni.edu.vn (N.T.A.N.); 4 Institute of Natural Products Chemistry, Vietnam Academy of Science and Technology (VAST), 18 Hoang Quoc Viet, Cau Giay District, Hanoi 122100, Vietnam; tranquoctoan2010@gmail.com (Q.-T.T.); dophuongthuy303@gmail.com (T.-T.D.); 5 Faculty of Hi-Tech Agricultural and Food Sciences, Dong A University, Da Nang City 550000, Vietnam; tmhung801018@gmail.com; 6 Institute of Applied Science and Technology, Van Lang University, Ho Chi Minh City 700000, Vietnam; hien.pp@vlu.edu.vn; 7 Department of Natural Sciences & Technology, Tay Nguyen University, Buon Ma Thuot, Dak Lak 630000, Vietnam; ntnhan@ttn.edu.vn (T.-N.N.); phantuquy@ttn.edu.vn (P.-T.Q.); 8 College of Agriculture and Forestry, Thai Nguyen University (TUAF), Quyet Thang 24119, Vietnam; nguyenthehung@tuaf.edu.vn (T.-H.N.); nguyenhuutho@tuaf.edu.vn (H.-T.N.); 9 Institute of Forestry Researh and Development, TUAF, Quyet Thang 24119, Vietnam

**Keywords:** *Tetradium ruticarpum*, tyrosine phosphatase 1B (PTP1B), α-glucosidase (3W37), molecular docking simulation

## Abstract

Folk experiences suggest natural products in *Tetradium ruticarpum* can be effective inhibitors towards diabetes-related enzymes. The compounds were experimentally isolated, structurally elucidated, and tested in vitro for their inhibition effects on tyrosine phosphatase 1B (PTP1B) and α-glucosidase (3W37). Density functional theory and molecular docking techniques were utilized as computational methods to predict the stability of the ligands and simulate interaction between the studied inhibitory agents and the targeted proteins. Structural elucidation identifies two natural products: 2-heptyl-1-methylquinolin-4-one (**1**) and 3-[4-(4-methylhydroxy-2-butenyloxy)-phenyl]-2-propenol (**2**). In vitro study shows that the compounds (**1** and **2**) possess high potentiality for the inhibition of PTP1B (IC_50_ values of 24.3 ± 0.8, and 47.7 ± 1.1 μM) and α-glucosidase (IC_50_ values of 92.1 ± 0.8, and 167.4 ± 0.4 μM). DS values and the number of interactions obtained from docking simulation highly correlate with the experimental results yielded. Furthermore, in-depth analyses of the structure–activity relationship suggest significant contributions of amino acids Arg254 and Arg676 to the conformational distortion of PTP1B and 3W37 structures overall, thus leading to the deterioration of their enzymatic activity observed in assay-based experiments. This study encourages further investigations either to develop appropriate alternatives for diabetes treatment or to verify the role of amino acids Arg254 and Arg676.

## 1. Introduction

**Figure 1:**
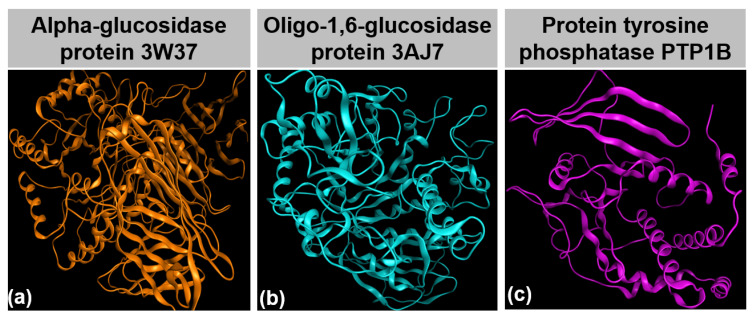
(**a**) α-glucosidase protein 3W37; (**b**) Oligo-1,6-glucosidase protein 3AJ7; (**c**) Protein tyrosine phosphatase 1B.

Diabetes mellitus (DM) is on the rise in the public. It has steadily become prevalent worldwide, most markedly in middle-income countries. The World Health Organization (WHO) estimated that diabetes was directly responsible for about 1.6 million deaths in 2015 and predicted that the increasing death toll would lead to the disorder becoming the seventh leading cause of death by 2030 [1]. Furthermore, evidence has suggested a link between diabetes and the cause of cardiovascular disease, blindness, kidney failure, stroke, and limb amputation [2]. In particular, type 2 DM, known as a non-insulin-dependent disorder, results from ineffective use of insulin in the body, accounting for 90–95% of total DM sufferers [3]. Therapeutic treatments for type 2 diabetes mainly relate to inhibition of insulin- and glucose-based enzymes. The former attempts to compensate for defects in insulin secretion and insulin action by approaches for insulin signaling regulation; meanwhile, the latter is used for controlling postprandial hyperglycemia by mitigating the activity of glucosidases, thus reducing gut glucose absorption [4]. First, protein tyrosine phosphatase (PTP1B) is a major glucose-homeostasis and energy-metabolism regulator, thus regarded as an attractive drug target for therapeutic intervention in type 2 diabetes and obesity [5]. In detail, it blocks insulin receptor substrate-1 and dephosphorylates phosphotyrosine residues, thus causing insulin insensitivity or even a cut-off of intracellular insulin signaling; meanwhile, regarding the leptin signaling pathway, PTP1B binds and dephosphorylates leptin receptor Janus kinase 2 (JAK2), thereby causing the malfunctioning of energy balance to emerge [6]. PTP1B structure was already well determined, and information on it is publicly available at UniProtKB, archived under entry ID: UniProtKB-A0A0U1XP67. Second, α-glucosidase, an exoenzyme found in animal, plant, bacterial, and fungual organisms, breaks down starch and disaccharides to glucose [7]. A study suggested that the enzyme can only yield the monosaccharides by catalyzing the hydrolysis of α-(1→4) and α-(1→6) bonds [8], confirming the sources of α-glucosidase from sugar beet seeds [9]. Protein structural data of the enzyme can be referenced at Worldwide Protein Data Bank under entry PDB-3W37. Third, oligo-1,6-glucosidase, often called isomaltase, is a debranching endoenzyme, hydrolyzing only the α-1,6 linkage in starch and glycogen to produce sugars with an α-configuration [10]. They cannot break the α-1,4 linkage. Although some bacterial species, such as *Bacillus cereus*, can synthesize oligo-1,6-glucosidase, this enzyme is present mainly in the animal kingdom [11]. In humans, it is located on the small intestine brush border [12]. Information on isomaltase crystal structure can be downloaded directly from Worldwide Protein Data Bank database under entry PDB-3AJ7. Therefore, PTP1B, 3W37, and 3AJ7 (Figure 1) are considered highly promising drug targets for the effective treatment of type 2 diabetes. In principle, multiple inhibitions of PTP1B and glycoside hydrolase proteins are a promising strategy to simultaneously suppress hyperglycemia and improve insulin sensitization.

*Tetradium ruticarpum* (A. Juss.) T.G. Hartley (Ngô thù, Xà lạp in Vietnamese) is a flowering plant in the Rutaceae family. This plant, previously called *Euodia ruticarpa* (*Wu Zhu Ru* in Chinese and *Goshuyu* in Japanese), is a species of deciduous, fruit-bearing tree in genus *Tetradium*. The other synonym names were *Ampacus ruticarpa* (A. Juss.) Kuntze, and *Evodia ruticarpa* (A. Juss.) Hook. F. and Thomson, they are found widely in North India and China. Both the former genus name and the species name are often misspelled, and the plant usually appears in sources dealing with traditional Chinese medicine as “*Evodia€ rutaecarpa*”, and thus, *Evodia* *rutaecarpa* is presently the most used name in the herbal list in several countries. This species (A. Juss.) was first described by T.G. Hartley in the literature in 1981 [13]. In Vietnam, the tree grows widely in Pho Bang, Ha Giang province in the North of Vietnam, and is also grown in several medicinal gardens [14]. Its fruit (*Fructus Tetradii Rutaecarpi*) has been clinically used to relieve several irregular symptoms such as headache, vomit, diarrhea, abdominal pain, dysmenorrhea, and pelvic inflammation for thousands of years in Traditional Chinese Medicine and also was used as an herbal medicine for centuries in East Asia, including Japan, Korea, Thailand, and Vietnam. Several secondary metabolites were isolated from this plant such as alkaloids, terpenoids, flavonoids, steroids, and phenylpropanoids, which demonstrated the effects on cancer, inflammation, cardiovascular diseases, and bacterial infection [15]. *T. ruticarpum* and its ingredients also exhibited pharmacological effects on obesity and diabetes. The hot water extract from *E. rutaecarpa* collection in Japan and its active compound, rhetsinine, inhibited aldose reductase in a dose-dependent manner, and aldose reductase inhibitors have been considered as having potential for the treatment of diabetic complications. In particular, rhetsinine inhibits sorbitol accumulation by 79.3% at 100 μM. Discussion of the results showed that rhetsinine might be potentially useful in the treatment of diabetic complications [16]. Moreover, a major quinolone alkaloid compound, evodiamine, extracted from *E. rutaecarpa* reduced obesity and insulin resistance in obese/diabetic KK-Ay mice via signal [17], suggesting the improvement of glucose tolerance and prevention of the progress of insulin resistance associated with obese/diabetic through inhibition of mTOR-S6K signaling and IRS1 serine phosphorylation in adipocytes for this compound. Furthermore, evodiamine was also found to activate peroxisome proliferator-activated receptors (PPARs), ligand-activated transcription factors that are involved in regulating glucose and lipid homeostasis, which were considered as a potential therapeutic target in Type 2 diabetes [18].

**Figure 2:**
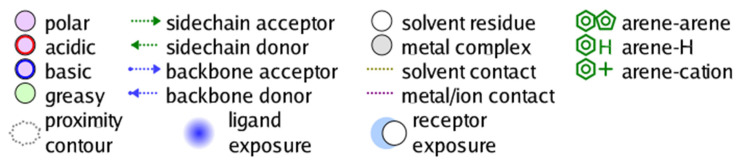
Descriptive denotation for in-pose interactions projected by MOE2015.10 molecular docking simulation.

In silico techniques based on computational simulation and computing calculation are currently seeing a gain in popularity in medical science as prescreening research. They reduce the cost and time of wet laboratory experiments by predicting the compounds with undesirable properties and the most promising candidates. The former substances are deemed to be eliminated from the next analysis or further-developed research, while the latter justify the selection. Regarding ligand–protein interaction, molecular docking simulation is an effective method to investigate the potency of a ligand as an inhibitor towards its targeted protein. The method can estimate ligand–target binding energy and intermolecular interaction, thus predicting the static stability of the inhibitory systems. Effectively inhibited by external ligands, the enzyme is likely to undergo conformational changes and thus the inevitably ensuing loss of enzymatic functionality. This might help to mitigate the amount of glucose catalytically synthesized and excreted into the bloodstream. Regarding ligand–protein inhibition simulated by molecular docking simulation, an associated docking score (DS) under −3.2 kcal·mol^−1^ indicates good binding capacity [19,20,21,22]. In principle, the figure is calculated by a sum of all intermolecular-interaction free energy. In particular, the affinity stems from hydrophilic bonding, i.e., various hydrogen-bond types, and hydrophobic binding, i.e., van der Waals forces. Furthermore, a root-mean-square deviation (RMSD) measures the average distance between internal atoms of an inhibitory system. Inhibition failure is proposed if this value is over 3 Å; meanwhile, docking success is justified by its associated RMSD value ≤ 2 Å [23]. In addition, inhibitory morphology and in-pose interaction are visually illustrated. Descriptive symbols regulated by MOE2015.10 are given in Figure 2.

In this study, a search for the anti-diabetic agents from Vietnamese medicinal plants was carried out. Two natural products were isolated from *T. ruticarpum*, structurally elucidated, and in vitro tested for their inhibition effects (on tyrosine phosphatase and α-glucosidase) by assay-based experiments. Density functional theory and molecular docking technique were utilized to predict the stability of the ligands and simulate the interaction between the studied inhibitory agents and the targeted proteins (3W37, 3AJ7, and PTP1B).

## 2. Results and Discussion

### 2.1. Experimental Results 

**Figure 3:**
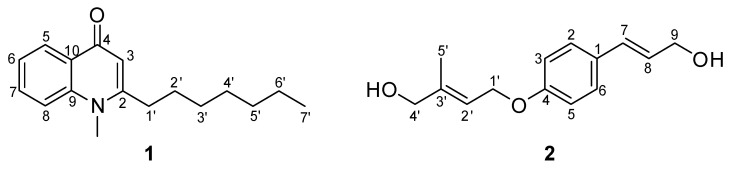
Chemical structure of isolated compounds **1** and **2** from *T. ruticarpum*.

The fruit of *T. ruticarpum* was extracted with MeOH and then partitioned with ethyl acetate to obtain an EtOAc fraction. By using liquid–liquid partition and column chromatographic separation, compounds **1** and **2** were purified from this EtOAc fraction. All the elucidated structural formulae are presented in Figure 3. 

**Figure 4:**
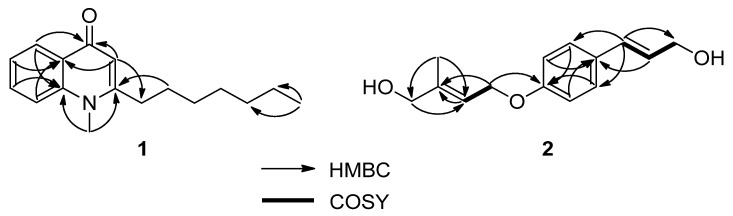
HMBC and COSY correlations of compounds **1** and **2** from *T. ruticarpum*.

Compound **1** was obtained as a white crystal and showing a positive reaction with Dragendorff’s reagent test. The molecular formula of **1** was determined to be C_17_H_23_NO based on the ion at *m*/*z* 258.1 [M + H]^+^ in the fast atom bombardment mass spectrometry (FAB-MS). The presence of an *N*-methyl group [*δ*_H_ 3.72 (3H, s)/*δ*_C_ 34.3], an olefinic group (*δ*_H_ 6.21 (1H, s, H-3)/*δ*_C_ 154.9 (C-2) and 111.3 (C-3)), a benzene ring (*δ*_H_ 8.43 (2H, dd, *J* = 8.0, 1.6 Hz, H-5), 7.36 (1H, brt, *J* = 8.0 Hz, H-6), 7.64 (1H, dt, *J* = 1.6, 8.0 Hz, H-7), and 7.49 (1H, brd, *J* = 8.0 Hz, H-8)/*δ*_C_ 126.8 (C-5), 123.4 (C-6), 132.2 (C-7), 115.5 (C-8), 142.1 (C-9), and 126.7 (C-10)), and a ketone group at *δ*_C_ 178.0 (C-4) in the ^1^H and ^13^C NMR spectra indicated **1** to be *N*-methyl-4(1*H*)-quinolone type alkaloids (Figure 3 and Supplementary data) [24,25]. The ^1^H NMR spectrum of **1** showed a signal of a heptyl moiety (2.69 (2H, t, *J* = 7.6 Hz, H-1′), 1.67 (2H, q, *J* = 7.6 Hz, H-2′), 1.27-1.42 (8H, m, H-3′/H-4′/H-5′/H-6′), 0.88 (3H, t, *J* = 6.8 Hz, H-7′)), indicating the C7 side chain of **1** (Figure 1). The ^13^C NMR and heteronuclear multiple quantum correlation (HMQC) spectra of **1** showed 17 carbon signals, including one *N*-methyl carbon at *δ*_C_ 34.3 (*N*-CH_3_) and seven carbon signals of a heptyl group (*δ*_C_ 34.9 (C-1′), 28.7 (C-2′), 29.4 (C-3′), 29.2 (C-4′), 31.8 (C-5′), 22.8 (C-6′), and 14.2 (C-7′) (Figure 1 and Supplementary data)). The HMBC correlations of *N*-CH_3_ to C-2 and C-9; H-3 to C-4 and C-10; H-5 to C-4, C-7, and C-9, as well as H-8 to C-6 and C-10, were observed, indicating the quinoline moiety of **1**. Furthermore, the location of the heptyl group was located at C-2 based on the HMBC correlations from H-3 to C-1′ and H-2′ to C-2 (Figure 4 and Supplementary data). Based on the above evidence and in the comparison with the published data, compound **1** was identified as schinifoline [25]. This compound **1** belongs to the alkylquinolin-4(*1H*)-one type alkaloid derived in the Rutaceae species and was first identified from *Zanthoxylum schinifolium* [25]. This study has presented for the first time the purification of natural schinifoline from *T. ruticarpum*. 

Compound **2** was obtained as colorless needles. The ^1^H NMR and COSY spectra of **2** showed signals of a 1,4-disubstituted benzene ring (*δ*_H_ 77.32 (2H, d, *J* = 8.4 Hz, H-2/H-6) and 6.87 (2H, d, *J* = 8.4 Hz, H-3/H-5)), assigned to an A_2_B_2_-spin system and a *trans*-3-hydroxy-1-propenyl group [(*δ*_H_ 6.55 (1H, brd, *J* = 16.0 Hz, H-7), 6.21 (1H, dt, *J* = 16.0, 6.0 Hz, H-8), and 4.30 (2H, dd, *J* = 6.0, 1.2 Hz, H-9)) (Figure 1). The signals at *δ*_H_ 4.59 (2H, brd, *J* = 6.4 Hz, H-1′), 5.77 (1H, m, H-2′), 4.09 (2H, s, H-4′), and 1.77 (3H, s, H-5′) in the ^1^H NMR spectrum were arranged to be the signal for 4-methylhydroxy-2-butenyl group (see Supplementary data). The cross-peak between H-1′ and H-2′ in the COSY spectrum further confirmed the existence of 4-methylhydroxy-2-butenyl group (Figure 3 and Supplementary data). The ^13^C NMR, distortionless enhancement by polarization transfer (DEPT) and heteronuclear single quantum correlation (HSQC) spectra of **2** showed the presence of 14 signals, including six carbons for the benzene ring (*δ*_C_ 129.5 (C-1), 127.6 (C-2/C-6), 114.9 (C-3/C-5), and 158.4 (C-4)), three carbon signals for a *trans*-3-hydroxy-1-propenyl group (*δ*_C_ 130.9 (C-7), 126.2 (C-8), and 63.9 (C-9)), and five carbon signals for a 4-methylhydroxy-2-butenyl group (*δ*_C_ 64.3 (C-1′), 119.7 (C-2′), 140.1 (C-3′), 67.8 (C-4′), and 14.0 (C-5′)). The correlations of H-7 to C2 and C-6; H-8 to C-1; H-5′ to C-2′, C-3′, and C-4′, as well as H-2/H-6 and H-1′ to C-4, in the HMBC spectrum confirmed the structure of compound **2** (Figure 4 and Supplementary data). The FAB-MS of compound **2** showed the *pseudo*-molecular ion at *m*/*z* 257.03 [M + Na]^+^, indicating the molecular formula of C_14_H_18_O_3_. Based on this observation, together with the comparison with the published data [25], compound **2** was identified as intergrifoliodiol. To the best of our knowledge, this compound has been isolated from *T. ruticarpum* for the first time in the literature, given our reachable referencing.

Genetic and biochemical studies have shown that PTP1B is a key negative regulator of leptin and insulin signaling pathways which are responsible for glucose homeostasis, control body weight, and energy expenditure [26]. This reveals the potential use of natural products with PTP1B activity inhibition or gene expression reduction effects for treating type 2 diabetes and obesity [3]. Our in vitro study showed that compounds **1**–**2** potentially inhibited the PTP1B enzyme activity with IC_50_ values of 24.3 ± 0.8 and 47.7 ± 1.1 μM, respectively (Table 1). Ursolic acid, a characteristic ursane-type triterpenoid mainly found in persimmon, was used as a positive control showing the IC_50_ value of 3.5 ± 0.3 μM. Furthermore, schinifoline (**1**) exhibited a potential inhibitory effect on α-glucosidase enzyme activity with an IC_50_ value of 92.1 ± 0.8 μM, two times stronger than acarbose, the positive control used for this assay having an IC_50_ value of 152.4 ± 0.6 μM. Integrifoliodiol (**2**) showed similar activity with acarbose, whose IC_50_ value was 167.4 ± 0.4 μM. 

The experimental assays reveal that **1** and **2** possess good inhibition activity towards α-glucosidase and protein PTP1B, given by their significant IC_50_ value in comparison to those of the control drugs. In detail, the corresponding values accord with the order **1** > **2** regarding either of the targeted proteins.

Schinifoline (SF) was known as a 4-quinolinone alkaloid and possessed some activities. Lu et al. reported that schinifoline inhibited the negative effects of *Candida albicans* in vivo by regulating lysosomal pathway-related genes that accelerate the metabolism and degradation of abnormal proteins [27]. In addition, schinifoline (**1**) and intergrifoliodiol (**2**) did not show in vitro inhibitory effect on nitric oxide production [28]. Wang et al. investigated the radio-sensitizing effect of schinifoline on A549 cells. The cell viability results indicated cytotoxicity of SF on A549 cells with IC_50_ values of 33.7 ± 2.4, 21.9 ± 1.9, and 16.8 ± 2.2 μg/mL after 6, 12, and 24 h treatment, respectively, with different concentrations. The results of cell proliferative inhibition and clonogenic assay showed that SF enhanced the radio-sensitivity of A549 cells when applied before ^60^Co γ-irradiation, and this effect was mainly time- and concentration-dependent [29]. Other studies showed that schinifoline have no antibacterial activity on *Staphyloccocus aureus* (ATCC25923), *Staphylococcus epidermidis* (ATCC12228), and *Bacillus subtilis* (ATCC6633) and no cytotoxicity against four human cancer cell lines (HepG2, Hela, BEL7402, and BEL7403) [30], nor Jurkat T cells clone E6.1 [31], using the MTT method. Furthermore, SF can be used to treat experimental liver cancer cells in rats by influencing cytoskeleton [32] and can treat experimental hepatocarcinogenesis by inhibiting hepatoma cell’s DNA synthesis and preventing the cytodiaeresis [33]. Although schinifoline (**1**) and integrifoliodiol (**2**) possessed some biological activities, their PTP1B inhibitory activity has not been investigated to date. Our study showed that compounds **1** and **2** potentially inhibited the PTP1B enzyme activity with IC_50_ values of 24.3 ± 0.8 and 47.7 ± 1.1 μM, respectively. This is the first time that compound **2** has been identified from *T. ruticarpum* and the PTP1B inhibitory activity of these compounds has been investigated, given our reachable referencing.

### 2.2. Computational Results

**Figure 5:**
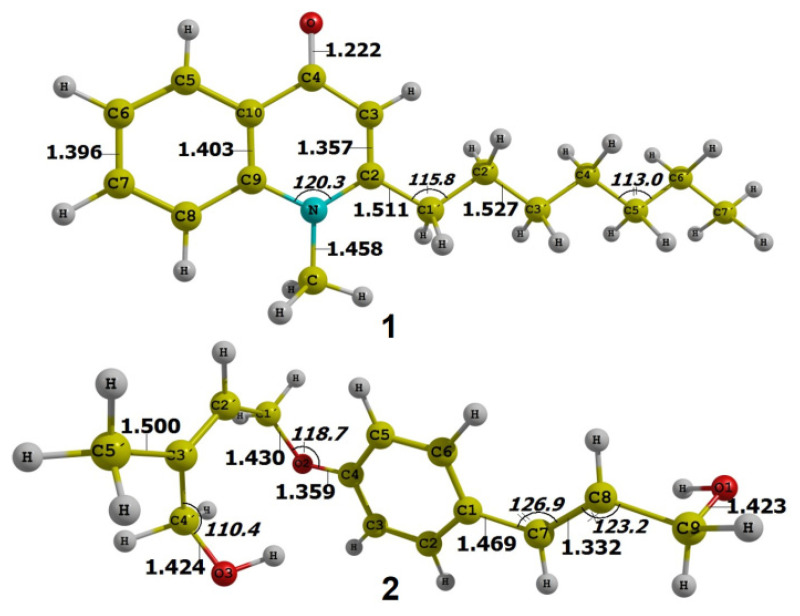
Optimized structures of the isolated compounds **1** and **2** calculated by DFT using basis M052X/6-311++G(d,p).

**Figure 6:**
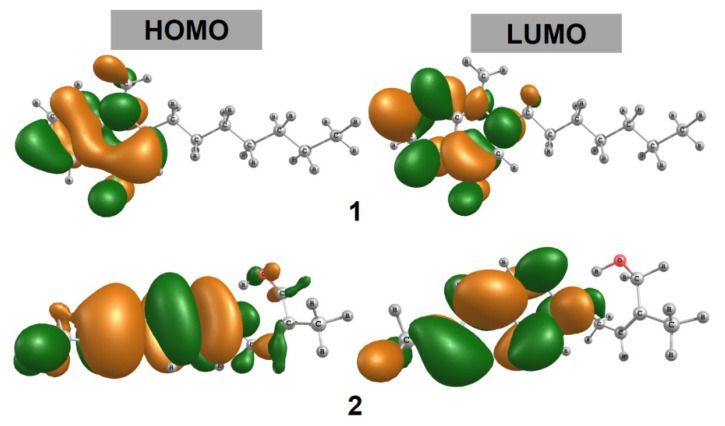
HOMO and LUMO of isolated compounds **1** and **2** in *T. ruticarpum* calculated by DFT at the level of theory M052X/def2-TZVP. The different colours (orange and green) refer to the deformation of electron densities, i.e. from green to orange. Nevertheless, an in-depth disscusion is unnecessary for quantum-unrelated analysis.

Geometrically optimized structures of **1**–**2** are shown in Figure 5, their frontier molecular orbitals (HOMO and LUMO) are presented in Figure 6, and the related quantum parameters are summarized in Table 2. Firstly, it can be noticed that **1** contains carbonyl and N-heterocyclic groups, while **2** is functionalized with groups -OH and C-O-C. These were reported to be highly conducive to polarity and solubility of the host compound [34,35], thus implying promising inhibitability towards protein molecules based on polar interactions with highly polarized amino acids. Although C-O-C might contribute insignificantly to the compound polarity [36], their well-demonstrated antitumoural activity still incentivizes the use for cytological applications [37,38]. Furthermore, bonding analysis on frontier molecular orbitals suggests that the compounds are suitable for intermolecular inhibition. In fact, electrons of their HOMO and LUMO are densely distributed and largely space-occupying in a certain region of the molecules, mainly localizing at those of cyclic groups. This indicates that the molecules are able to initiate intermolecular inhibition from certain approaching manners that uphold electron-transferring interactability. In addition, there are no significant differentials between the parameter values calculated for each compound. In particular, the energy gaps (Δ*E_GAP_*) of 1 and 2 are −6.884 and −7.102 eV, respectively. These are considered relatively low values and thus likely conducive to chemical reactivity and inhibitory stability [39,40]. The reason is thought to be that electrons of inhibitor molecules are easily activated and transferred to their surface, ready for intermolecular activities. Furthermore, electronegativity (*χ*), or the chemical potential (*μ*) in a negative value, could be considered as a reliable inhibition indicator since it presents an electron-attracting tendency. In principle, a higher electronegativity implies a stronger attraction of electrons towards the host molecule. Therefore, the compounds seem promising for docking investigation.

**Figure 7:**
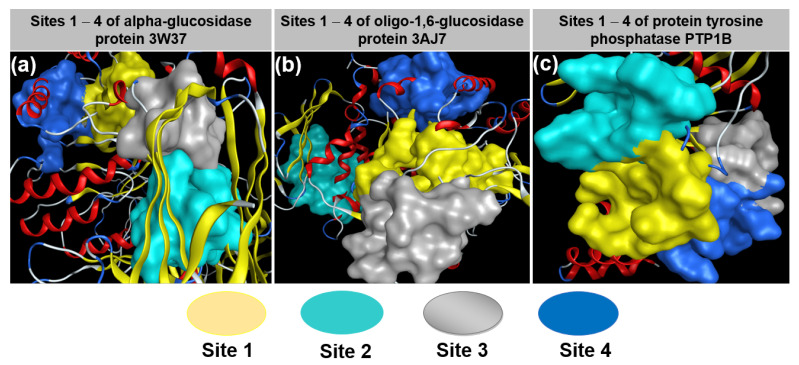
Quaternary structures of proteins (**a**) 3W37, (**b**) 3AJ7, and (**c**) PTP1B with their approachable sites by investigated compounds **1** and **2**: site 1 (yellow), site 2 (cyan), site 3 (grey), and site 4 (blue).

The quaternary structure of the targeted proteins 3W37 (also known as α-glucosidase), PTP1B (also known as tyrosine phosphatase 1B), 3AJ7 (oligo-1,6-glucosidase), and their approachable sites by investigated compounds **1** and **2** are virtually represented in Figure 7. The corresponding in-pose amino acid residues are listed in Table 3. The results of prescreening on the inhibitability of compounds **1** and **2** (and a commercialized drug voglibose) towards these potential sites are summarized in Table 4. There are four sites found for the investigated compounds to entry, assigned as site 1 (yellow), site 2 (cyan), site 3 (grey), and site 4 (blue). All sites comprise large numbers of different amino acids, meaning that they can be considered highly conducive to peripheral interactions. Significantly, site 2 of protein 3W37 comprises 21 different amino acids, a predominant number in comparison to others, implying its most effective inhibitability. In fact, prescreening results also indicate that it is the most active site for the investigated compounds given by the lowest DS values (varying from −13.2 to −14.1 kcal·mol^−1^) and the number of interactions created (at least 3). Regarding protein PTP1B, there are no noticeable differences between the corresponding numbers of its detected sites. The lowest DS value and the corresponding number of interactions are found at site 1. In terms of 3AJ7, sites 1 and 3 are expected, providing better inhibitability than the others due to their dominant numbers of in-pose amino acids, i.e., 36 and 23, respectively. They are also the most effective inhibitory regions for **2** and **1**, respectively. In addition, the comparable equivalency of the figures in comparison to those of the commercialized drug voglibose (**D**) justifies their inhibitory potentiality towards these diabetes-related proteins. Therefore, these sites opt for more in-depth analysis in the attempt to retrieve a structure–activity relationship between experiment-based and computer-based research studies.

The detailed docking simulation results for the inhibitory duos are summarized in Table 5. Overall, both compounds exhibit better inhibitability towards PTP1B than towards the carbohydrate-hydrolases (3W37 and 3AJ7). In fact, DS values obtained from calculations for **1-PTP1B** and **2-PTP1B** inhibitory complexes are −14.9 and −14.7 kcal·mol^−1^, markedly lower than those of **1-3W37** and **2-3W37**, i.e., −13.2 and −13.8 kcal·mol^−1^, respectively. The former also contains more either hydrophilic or hydrophobic interactions between the ligands and the in-pose amino acids than the latter. In particular, the PTP1B-based inhibitions are formed by five hydrogen bond interactions and eight or nine van de Waals interactions; meanwhile, the corresponding figures for 3W37-based inhibitory systems are three and nine, respectively. All the inhibitory systems are considered biologically rigid since their RMSD value are all under 2 Å. Furthermore, the computed data regarding oligo-1,6-glucosidase (**1-3AJ7** and **2-3AJ7**) are included for further reference as the enzyme is well-known as an important target in diabetes-related research, despite the fact that it is not included in the experimental section in this study. These altogether can explain the experimental results that the studied compounds, i.e., **1** and **2**, can inhibit enzyme tyrosine phosphatase 1B at significantly lower IC_50_ values than the carbohydrate-hydrolase counterpart. Although the primary parameters extracted from computational output (i.e., DS value and number of interactions) are highly consistent with the overall inhibition activity towards the enzymes (i.e., tyrosine phosphatase 1B and α-glucosidase) obtained from experimental assays, the specific orders regarding each of these proteins are still not clearly represented.

Nevertheless, a brief structure relationship for the inhibition strength of the isolated compounds **1** and **2** from *T. ruticarpum* on each enzyme can be reached based on certain hydrogen bonding interactions. First, the docking simulation technique expects that there exists a strong hydrogen bond formed between an oxygen atom of the ligand **1** and a nitrogen atom of amino acid Arg254 within the distance 2.92 Å in **1-PTPB1B**. The bonding registers an exceptionally high value of Gibbs free energy −4.4 kcal·mol^−1^. While there are five predicted hydrogen bonds that contribute to the formation of the inhibitory system **2-PTPB1**, the amino acid is not involved. This highly correlates with the results obtained from the enzyme assays on tyrosine phosphatase 1B inhibition; i.e., **1** (IC_50_ 24.3 ± 0.8 µM) > **2** (IC_50_ 47.7 ± 1.1 µM). Similarly, amino acid Arg676 is likely to play an important role in the formation of **1-3W37** inhibitory complex as it interacts with the ligand via three hydrogen-bonding interactions, whose Gibbs free energy in total is −5.9 kcal·mol^−1^. Meanwhile, **2** only creates one of the hydrophilic bonding with its targeted protein to form **2-3W37** inhibitory structure. The value of −2.5 kcal·mol^−1^ corresponds to the free Gibbs energy of this interaction. This can also be related to the differences observed between the enzyme assays on α-glucosidase inhibition. Therefore, the sufficient binding carried out on these amino acids, i.e., Arg254 and Arg676, seems to induce serious conformational changes on the enzymes PTP1B and 3W37, respectively, thus leading to the loss of their enzymatic functionality that is experimentally demonstrated in enzyme assays by the increase in IC_50_ values. In terms of oligo-1,6-glucosidase-based inhibitory systems (**1-3AJ7** and **2-3AJ7**), the concern is thought to relate to amino acid Arg442. The intuitive speculation still requires further relevant experimental work specifically targeting these amino acids to verify the proposed inhibition models.

**Figure 8:**
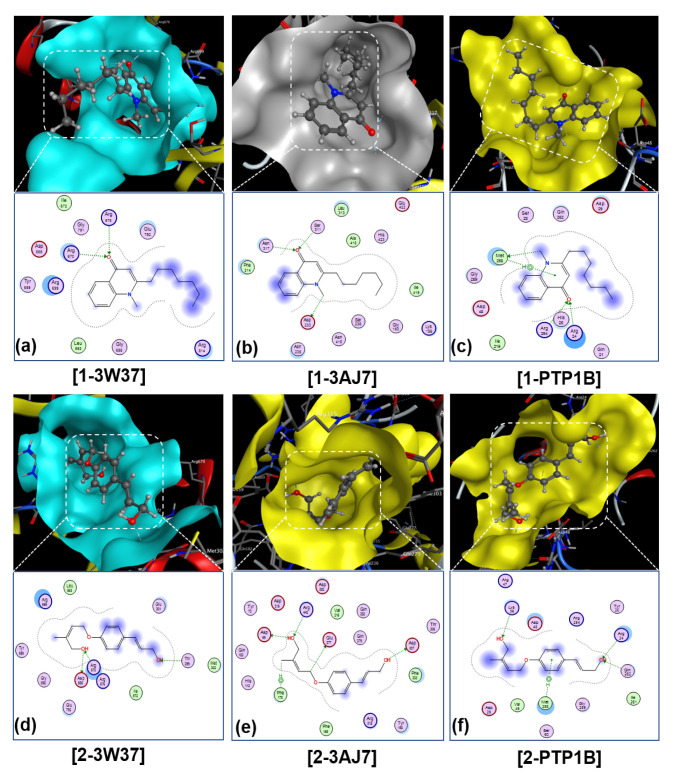
Visual presentation and in-pose interaction map of ligand-3W37, ligand-3AJ7, and ligand-PTP1B inhibitory isolated compounds **1** and **2**: (**a**) **1-3W37**, (**b**) **1-3AJ7**, (**c**) **1-PTP1B**, (**d**) **2-3W37**, (**e**) **2-3AJ7**, (**f**) **2-PTP1B**. In molecular rendering, black, gray (white), blue, and red are standard colours for carbon, hydrogen, nitrogen, and oxygen atoms, respectively.

The inhibitory structures and their in-pose intermolecular interactions are visually shown in Figure 8. Although the sites are observably uncapacious for either macromolecule to enter or for simultaneous inhibitions, the investigated compounds are still predicted to geometrically fit in the structural topography of their inhibiting sites given their continuous proximity contours. This indicates a high degree of complementarity. In addition, the simulation for the commercialized drug voglibose (**D**) was also screened, and the corresponding results are included in supplementary data (Table S1 and Figure S16) for optional reference.

## 3. Materials and Methods

### 3.1. Experimental Methods

#### 3.1.1. General Experimental Procedures

Proton and carbon nuclear magnetic resonance (NMR) were measured on a Varian Unity Inova 400 and/or 500 MHz spectrometers. Mass spectrometry (MS) data were obtained from a Varian FT-MS spectrometer and (Bruker Daltonics, Ettlingen Germany). Normal-phase and reverse-phase silica gels (F_254_, 40–63 mesh) were purchased from Merck, St. Louis, MO, USA. NP and RP thin-layer chromatography (TLC) plates were from Merck (St. Louis, MO, USA). High-performance liquid chromatography (HPLC, Santa Clara, CA 95051, USA) was carried out using a 1260 Agilent HPLC System: G1311C pump, G2260A auto-sampler, G1316A Thermo, and G1315D detector. Optima_Pak C18 column (10 × 250 mm, 10 and/or 5 µm particle sizes), RS Tech, Korea, and/or Zorbax eclipse XDB-C18 (9 × 250 mm, 5 µm particle size) were used for purification. 

#### 3.1.2. Plant Material 

*Tetradium ruticarpum* material was obtained in 2019, and the plant sample was identified by Dr. Quoc-Binh Nguyen (Vietnam National Museum of Nature, Vietnam Academy of Science and Technology—VAST, Ha Noi, Vietnam). The specimen voucher of this plant was stored at INPC, VAST, Vietnam. 

#### 3.1.3. Isolation and Purification 

The buds (1.2 kg) were extracted with methanol (5.0 L × 3 times) at 45 °C using sonication for 5 h. After filtration, the extracted solution was then evaporated by a rotary system to remove the solvent. The total methanol crude extract obtained was further suspended in distilled water (1.0 L) and partitioned with EtOAc (1.0 L × 4 times). The resulting fractions were concentrated under reduced pressure in a rotary evaporator to give the EtOAc and an H_2_O residue, respectively. An activity-guided fractionation study resulted in the EtOAc extract being chosen for further study (Table S3 in Supplementary data).

The EtOAc fraction was thus directly chromatographed on an open silica gel column (5.0 × 60 cm; 63–200 μm particle size) using a stepwise gradient of hexane/acetone (from 20:1 to 0:1) to give ten fractions (TR-1 to TR-10). By the α-glucosidase activity-guided isolation (Table S3), fraction 2 (TR-2) was further selected for purification by diluting to methanol until saturation and then kept still for precipitation. The supernatant was then separated from the precipitate (TR-2P) and evaporated to give a crude fraction (TR-2S). These two sub-fractions further tested the alkaloid-containing compound by reaction with Dragendorff reagent on a TLC plate. Fraction TR-2S was prior purified by an open silica gel column (2.0 × 60 cm, 40–63 μm particle size) due to its alkaloid-containing fraction, eluted with a gradient solvent system of hexane/EtOAc (from 15:1 to 10:1), and afforded compounds **1** (23.9 mg) and **2** (19.5 mg). 

*Schinifoline (**1**)*: White crystal; FAB-MS *m*/*z:* 258.1 [M + H]^+^ (C_1__7_H_23_NO); ^1^H-NMR (400 MHz, CDCl_3_) *δ*_H_ (ppm): 6.21 (1H, s, H-3), 8.43 (2H, dd, *J* = 8.0, 1.6 Hz, H-5), 7.36 (1H, brt, *J* = 8.0 Hz, H-6), 7.64 (1H, dt, *J* = 1.6, 8.0 Hz, H-7), 7.49 (1H, brd, *J* = 8.0 Hz, H-8), 2.69 (2H, t, *J* = 7.6 Hz, H-1′), 1.67 (2H, q, *J* = 7.6 Hz, H-2′), 1.27-1.42 (8H, m, H-3′/H-4′/H-5′/H-6′), 0.88 (3H, t, *J* = 6.8 Hz, H-7′), 3.72 (3H, s, N-CH_3_); ^13^C-NMR (100 MHz, CDCl_3_) *δ*_C_ (ppm): 154.9 (C-2), 111.3 (C-3), 178.0 (C-4), 126.8 (C-5), 123.4 (C-6), 132.2 (C-7), 115.5 (C-8), 142.1 (C-9), 126.7 (C-10), 34.9 (C-1′), 28.7 (C-2′), 29.4 (C-3′), 29.2 (C-4′), 31.8 (C-5′), 22.8 (C-6′), 14.2 (C-7′), 34.3 (N-CH_3_). 

*Integrifoliodiol(**2**)*: Colourless needles; FAB-MS *m*/*z:* 257.03 [M + Na]^+^ (C_1__4_H_1__8_O_3_); ^1^H-NMR (400 MHz, CDCl_3_) *δ*_H_ (ppm): *δ*_H_: 7.32 (2H, d, *J* = 8.4 Hz, H-2/H-6), 6.87 (2H, d, *J* = 8.4 Hz, H-3/H-5), 6.55 (1H, brd, *J* = 16.0 Hz, H-7), 6.21 (1H, dt, *J* = 16.0, 6.0 Hz, H-8), 4.30 (2H, dd, *J* = 6.0, 1.2 Hz, H-9), 4.59 (2H, brd, *J* = 6.4 Hz, H-1′), 5.77 (1H, m, H-2′), 4.09 (2H, s, H-4′), 1.77 (3H, s, H-5′); ^13^C-NMR (100 MHz, CDCl_3_) *δ*_C_ (ppm): 129.5 (C-1), 127.6 (C-2/C-6), 114.9 (C-3/C-5), 158.4 (C-4), 130.9 (C-7), 126.2 (C-8), 63.9 (C-9), 64.3 (C-1′), 119.7 (C-2′), 140.1 (C-3′), 67.8 (C-4′), 14.0 (C-5′).

#### 3.1.4. Protein Tyrosine Phosphatase 1B Inhibition Assay

Protein tyrosine phosphatase 1B (human recombinant), i.e., PTP1B, was purchased from Biomol International LP, Plymouth Meeting, Pennsylvania, PA, USA, and the inhibitory activities of the tested samples were evaluated using the method as described. [26] In a typical procedure, 0.05–0.1 μg of PTP1B (BIOMOL International L.P., Plymouth Meeting, Pennsylvania, PA, USA) and 4 mM *p*-NPP in a buffer containing 1 mM dithiothreitol, 0.1 M NaCl, 1 mM EDTA, and 50 mM citrate (pH 6.0), with or without test compounds, were added, with 100 μL final volume to each of the 96 wells. After the reaction mixture was incubated at 37 °C for 30 min, 10 M NaOH was added to quench the reaction. PTP1B enzyme activity was determined by the amount of produced *p*-nitrophenol at 405 nm. The nonenzymatic hydrolysis of the substrate was corrected by measuring the control, which contained no PTP1B enzyme. Ursolic acid (UA) was used as a positive control.

#### 3.1.5. α-Glucosidase Inhibition Assay

The inhibitory activity of *α*-glucosidase was determined according to the modified method with a readily available enzyme. In a typical procedure, *α*-glucosidase (0.1 U/mL, Wako) was dissolved in 100 mM sodium phosphate buffer (pH 6.8) and used as an enzyme solution. A total of 1 mM *p*-Nitrophenyl-*α*-D-glucopyranoside (*p*NPG) in the same buffer (pH 6.8) was used as a substrate solution. Then, 95 μL of enzyme solution, 10 μL of extracts, and 10 μL of ethanol and PBS solution (1:1) were mixed in the same 96-well plate for each concentration (250 μg/mL, 125 μg/mL, 62.5 μg/mL, 31.25 μg/mL) and measured absorbance at 405 nm after incubation for 5 min. After substrate solution (95 μL) was added, the sample solution was incubated for another 10 min at 37 °C incubator. Enzymatic activity was quantified by measuring absorbance at 405 nm using an enzyme-linked immunosorbent assay (ELISA) microplate reader (D.I Biotech Ltd., Seoul, Korea). The IC_50_ value was defined as the concentration of *α*-glucosidase inhibitor that inhibited 50% of *α*-glucosidase activity. Acarbose was used as a positive control, and all assays were conducted in triplicate.

#### 3.1.6. Statistical Analysis 

The collected data were analyzed using SPSS 20.0 software (SPSS Inc., Chicago, IL, USA). Average values and percentages were calculated. Differences in mean values were compared using analysis of variance (ANOVA), for normally distributed data, and the Mann–Whitney U test, for non-normally distributed data. Differences were considered significant at *p* < 0.05.

### 3.2. Computational Methods

#### 3.2.1. Quantum Chemical Calculation 

Density functional theory (DFT) was utilized to investigate quantum properties of the isolated compounds **1** and **2** from *T. ruticarpum*. Their molecular geometry was optimized using Gaussian 09 without symmetry constraints [41] at the level of theory M052X/6-311++G(d,p) [42]. Calculation of vibrational frequencies on the molecules was used to confirm that their structures were in global minimum on the potential energy surface (PES). Single-point energies at the M052X/6-311++G(d,p)-level-optimized geometries were calculated with the frozen-core approximation for non-valence-shell electrons by a larger basis set def2-TZVPP [43]. Each run of the optimization was based upon resolution-of-identity (RI) approximation. Frontier orbital analysis was implemented at the level of theory BP86/def2-TZVPP, providing localized molecular orbitals and orbital energy. NBO 5.1, available in Gaussian 09, was responsible for the analysis [44]. Bonding analysis revealed information of molecular electron density distribution. The highest occupied molecular orbital (HOMO) energy, *E_HOMO_*, represents intermolecular electron donation tendency; meanwhile, the electron-accepting ability of a molecule can be inferred from its value *E_LUMO_* (for lowest unoccupied molecular orbital—LUMO). Energy gap Δ*E* = *E_LUMO_* − *E_HOMO_* is considered as an indicator for intermolecular reactivity since it exhibits the formation of excited-state electrons towards the molecular surface. Ionization potential (*I*) and electron affinity (*A*) were calculated using Koopmans’ theorem [45], which negatively correlated with HOMO and LUMO energy as *I = −E_HOMO_* and *A = −E_LUMO_*. They then were used to yield the electronegativity (*χ*) of a molecule via the equation *χ* = (*I* + *A*)/2. Regarding an N-electron system with total electronic energy (*E*) and external potential *ν*(*r*), electronegativity (*χ*) is defined as the negative value of chemical potential (*μ*) [46,47]. This can be expressed by the following equation: *χ* = −*μ* = −(∂E/∂N)_*ν*__(*r*)_.

#### 3.2.2. Molecular Docking Simulation 

MOE 2015.10 software was responsible for molecular docking simulation. Its requisite input includes structural information of docking participants, i.e., two ligands **1**-**2**, PTP1B, and glycoside-hydrolase proteins (3W37 and 3AJ7). The simulated ligand-protein inhibitory structures were evaluated based on docking score (DS) energy as the main indicator. Root-mean-square deviation (RMSD) and intermolecular interactions were also analyzed and discussed. The typical procedure of a molecular docking simulation followed three steps [19,20,21,22]:

(a) Pre-docking preparation: Structural information of the targeted proteins can be referenced at UniProtKB and Worldwide Protein Data Bank: tyrosine phosphatase protein PTP1B (entry: UniProtKB—A0A0U1XP67), alpha-glucosidase protein 3W37 (DOI: 10.2210/pdb3W37/pdb), and oligo-1,6-glucosidase protein 3AJ7 (DOI: 10.2210/pdb3AJ7/pdb). The protein structure and their 3D protonation were prepared using Quickprep tool. Their active sites were determined based on a ligand–amino acid radius of 4.5 Å. The protein structures obtained were saved in format *.pdb. The investigated adducts were optimized based on the configuration: Conj Grad for minima energy; termination for energy change = 0.0001 kcal·mol^−1^; max interactions = 1000; modify charge: Gasteiger–Huckel.

(b) Docking investigation: After input preparation, intermolecular interaction simulation was performed on MOE 2015.10 system and simulated ligand–protein inhibitory structures were saved in format *.sdf. The docking simulation parameters were configured: poses retained for intermolecular interaction probing = 10; maximum solutions per iteration = 1000; maximum solutions per fragmentation = 200.

(c) Post-docking analysis: Docking score (DS) energy represents binding affinity of ligands and their targeted proteins in the site–site distance of a certain duo-system, thus considered as the primary indicator of inhibitability. Intermolecular interactions formed between the ligands and in-pose amino acids of the proteins included hydrophilic binding, e.g., electron-transferring (H-acceptor/donor), cation-arene (H-π), arene-arene (π-π), and ionic and hydrophobic interaction, also known as van der Waals forces. The simulation results in bonding amino acids, bonding lengths, and their Gibbs free energy regarding these interactions. Furthermore, static conformation of an inhibitory complex was predicted by its root-mean-square deviation (RMSD) value. This is based on the fact that RMSD represents the average between neighboring atoms; therefore, a smaller value means a more tightly bound conformation is formed. In addition, ligand conformation and orientation in its inhibited-protein active site were visualized on 2D and 3D planes. 

## 4. Conclusions

Two isolated natural compounds were successfully identified by structural elucidation as schinifoline (**1**) and integrifoliodiol (**2**). Enzyme assays demonstrated that the alkaloid compound (**1**) is the stronger inhibitor towards the both proteins tyrosine phosphatase 1B (PTP1B) and α-glucosidase (3W37). The corresponding orders are **1** (IC_50_ 24.3 ± 0.8 µM) > **2** (IC_50_ 47.7 ± 1.1 µM) regarding the former and **1** (IC_50_ 92.1 ± 0.8 µM) > **2** (IC_50_ 167.4 ± 0.4 µM) regarding the latter. The primary parameters obtained from molecular docking simulation, also known as DS value and number of interactions, confirm these experimental yields. In particular, in-depth analyses on structure–activity relationships imply that amino acids Arg254 and Arg676 might play an important role in the overall conformational changes of PTP1B and 3W37, respectively. This speculation still needs more specifically experimental work to verify, thus encouraging further continuous research from the readership. 

## Figures and Tables

**Table 1:** PTP1B and α-glucosidase inhibitory activities of compounds (**1**‒**2**) isolated from *T. ruticarpum*.

Compounds	PTP1B	α-Glucosidase
	IC_50_, µM ^a^	IC_50_, µM ^a^
**1**	24.3 ± 0.8	92.1 ± 0.8
**2**	47.7 ± 1.1	167.4 ± 1.4
Ursolic acid ^b^	3.5 ± 0.3	-
Acarbose ^b^	- ^c^	152.4 ± 0.6

^a^ Results are expressed as IC_50_ values (µM), determined by regression analysis and expressed as the means ± SD of three replicates. ^b^ Positive control. ^c^ Data not determined.

**Table 2:** Quantum chemical parameters of the isolated compounds **1**–**2** from *T. ruticarpum* calculated by NBO analysis at level BP86/def2-TZVPP including HOMO energy (*E_HOMO_*), LUMO energy (*E_LUMO_*), energy gap (Δ*E_GAP_*); ionization potential (*I*); electron affinity (*A*); electronegativity (*χ*); chemical potential (*μ*).

Compound	*E_HOMO_* (eV)	*E_LUMO_* (eV)	Δ*E_GAP_* = *E_LUMO_* − *E_HOMO_*	*I* = −*E_HOMO_*	*A* = −*E*_LUMO_	*χ* = (*I* + *A*)/2	*μ* = −*χ* = −(∂E/∂N)_*ν*(*r*)_
**1**	−7.238	−0.354	−6.884	7.238	0.354	3.796	−3.796
**2**	−7.374	−0.272	−7.102	7.374	0.272	3.823	−3.823

**Table 3:** In-site amino acid residues of proteins 3W37, 3AJ7, and PTP1B.

Site	Colour	Residues of Protein 3W37	Residues of Protein 3AJ7	Residues of Protein PTP1B
**1**	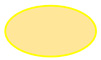	Tyr360 Phe367 Pro395 Ile396 Leu397 Ile454 Phe457 Arg458 Ile463 Ile466	Asp69 Tyr72 His112 Lys156 Ser157 Tyr158 Phe159 Leu177 Phe178 Gln182 Arg213 Asp215 Val216 Ser240 Ser241 Asp242 Glu277 Gln279 His280 Phe303 Thr306 Asp307 Thr310 Ser311 Pro312 Leu313 Phe314 Arg315 Tyr316 His351 Asp352 Gln353 Glu411 Ile440 Arg442 Arg446	Arg24 Ala27 Ser28 Asp29 Phe30 Pro31 Cys32 Lys36 Asp48 Val49 Phe52 Ile219 Arg254 Arg257 Met258 Gly259 Gln262
**2**	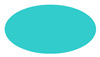	Glu301 Tyr659 Thr662 Leu663 Asp666 Arg670 Ile672 Arg676 Ile697 Gly698 Arg699 Gly700 Ile701 Ile754 Asn758 Ile759 Val760 Ala761 Thr790 Gly791 Glu792	Val369 Ile370 Lys373 Pro488 Asn489 Ser490 Asn493 Phe494 Glu497 Leu561 Glu562 Phe563 Gly564 Asn565 Tyr566 Pro567 Lys568 Val571	Ala35 Lys36 Leu37 Pro38 Asn40 Lys41 Asn44 Arg45 Tyr46 Arg47 Asp48 Val49 Ser50
**3**	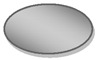	Tyr319 Pro658 Tyr661 Gln763 Arg773 Phe777 Leu793 Phe794 Leu795 Asp796 Trp841	Lys156 Ser157 Tyr158 Phe159 Gly160 Gly161 Asp233 Asn235 Ser236 Thr237 Trp238 Ser311 Leu313 Phe314 Asn317 Asn415 Ala418 Ile419 Glu422 His423 Glu428 Glu429 Lys432	Leu71 Lys73 Met74 Glu75 Ala77 Gln78 Arg79 Ser80 Ser203 Leu204 Ser205 Pro206 His208 Gly209 Pro210 Val211 Leu233 Lys237
**4**	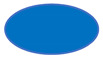	Tys331 Arg332 Asp333 Ile358 Asp359 Tyr360 Met361 Asp362 Ala363 Phe364 Asp370 His373 Phe374 Arg629	Lys13 Trp15 Asn259 Ile262 Glu271 Ile272 Met273 Thr274 Tyr289 Thr290 Ser291 Ala292 Arg294 His295 Glu296 Leu297 Ser298 Asp341 Cys342 Trp343	Lys73 Met74 Glu75 Glu76 Ala77 Thr230 Leu234 Lys248 Val249 Glu252 Lys255 Phe256

**Table 4:** Prescreening results on inhibitability of investigated compounds **1, 2** and the commercialized drug voglibose (**D**) towards the potential sites on proteins 3W37, 3AJ7, and PTP1B.

Compound	Protein 3W37								Protein 3AJ7								Protein PTP1B							
	Site 1		Site 2		Site 3		Site 4		Site 1		Site 2		Site 3		Site 4		Site 1		Site 2		Site 3		Site 4	
	E	N	E	N	E	N	E	N	E	N	E	N	E	N	E	N	E	N	E	N	E	N	E	N
**1**	−11.4	2	−13.2	3	−12.1	2	−10.9	3	−10.6	3	−11.4	2	−13.1	3	−11.7	3	−14.9	4	−12.4	3	−13.1	2	−11.8	3
**2**	−12.3	2	−13.8	3	−11.4	3	−11.7	2	−14.3	5	−12.8	3	−11.9	4	−11.3	3	−14.7	5	−13.4	4	−12.6	3	−11.4	4
**D**	−11.7	3	−14.1	4	−12.5	3	−10.6	3	−13.6	4	−10.4	3	−11.5	3	−12.4	4	−12.4	3	−14.5	4	−11.8	3	−10.7	3

**E**: DS value (kcal ∗ mol^−1^); **N**: Number of interactions.

**Table 5:** Molecular docking simulation results for inhibitory complexes between the ligands (compounds **1** and **2**) and the targeted proteins (3W37, 3AJ7, and PTP1B): **1-3W37**, **1-3AJ7**, **1-PTP1B**, **2-3W37**, **2-3AJ7**, and **2-PTP1B**.

Ligand-Protein Complex			Hydrogen Bond						Van der Waals Interaction
Name	DS	RMSD	L	P		T	D	E	
**1-3W37**	−13.2	1.84	O	N	Arg676	H-acceptor	3.46	−1.0	Arg814, Gly698, Leu663, Arg699, Tyr659, Asp666, Gly791, Ile672, Glu792
			O	N	Arg676	H-acceptor	3.02	−4.0	
			O	N	Arg676	H-acceptor	3.39	−0.9	
**1-3AJ7**	−13.1	1.33	C	O	Asp233	H-donor	2.91	−0.7	Asn235, Asn415, Ser236, Gly160, Lys156, Ile419, Phe314, Leu313, Ala418, His423, Glu422
			O	O	Ser311	H-acceptor	3.09	−1.3	
			O	N	Asn317	H-acceptor	2.94	−1.1	
**1-PTP1B**	−14.9	1.25	C	S	Met258	H-donor	3.83	−0.8	Ile219, Asp48, Gly259, Ser28, Gln262, Asp29, Gln21, His25
			O	N	Arg24	H-acceptor	3.09	−0.7	
			O	N	Arg254	H-acceptor	2.92	−4.4	
			6-ring	C	Met258	π-H	3.44	−1.6	
**2-3W37**	−13.8	1.07	O	O	Asp666	H-donor	2.89	−2.1	Glu792, Gly698, Tyr659, Arg699, Leu663, Glu301, Met302, Arg676, Ile672
			O	O	Thr299	H-acceptor	2.89	−1.3	
			O	N	Arg676	H-acceptor	3.18	−2.5	
**2-3AJ7**	−14.3	0.83	O	O	Asp307	H-donor	2.98	−1.2	Tyr72, Asp215, Asp362, Val216, Gln353, Gln279, Thr306, Phe303, Tyr158, Arg315, Phe159, His112,Gln182
			C	O	Glu277	H-donor	3.48	−0.6	
			O	O	Asp69	H-donor	2.73	−3.6	
			O	N	Arg442	H-acceptor	2.82	−4.3	
			C	6-ring	Phe178	H-π	3.69	−0.6	
**2-PTP1B**	−14.7	1.62	O	N	Arg24	H-acceptor	2.86	−2.6	Arg47, Asp48, Arg254, Tyr20, Ile261, Gly259 Ser50, Asp29, Val49
			O	N	Arg24	H-acceptor	3.07	−1.0	
			O	N	Gln262	H-acceptor	2.85	−0.8	
			O	N	Lys36	H-acceptor	3.27	−2.1	
			6-ring	C	Met258	π-H	4.20	−0.9	

**DS**: Docking score energy (kcal·mol^−1^); **RMSD**: Root-mean-square deviation (Å); **L**: Ligand; **P**: Protein; **T**: Type; **D**: Distance (Å); **E**: Energy (kcal·mol^−1^).

## Data Availability

The data presented in this study are available in this article.

## Supplementary Materials

The following are available online. Figure S1: ^1^H NMR spectrum of compound **1**, Figures S2 and S3: ^1^H NMR spectrum of compound **1** (expanded), Figure S4: ^13^C NMR spectrum of compound **1**, Figure S5: ^13^C NMR spectrum of compound **1** (expanded), Figure S6: HMQC spectrum of compound **1**, Figure S7: HMBC spectrum of compound **1**, Figure S8: Mass spectrum of compound **1**, Figure S9: ^1^H NMR spectrum of compound **2**, Figure S10: ^13^C NMR spectrum of compound **2**, Figure S11: DEPT spectrum of compound **2**, Figure S12: COSY spectrum of compound **2**, Figure S13: HSQC spectrum of compound **2**, Figure S14: HMBC spectrum of compound **2**, Figure S15: Mass spectrum of compound **2**, Figure S16: Visual presentation and in-pose interaction map of voglibose-protein inhibitory complexes: (a) D-3W37, (b) D-3AJ7, (c) D-PTP1B, Table S1: Molecular docking simulation results for inhibitory complexes between the controlled drug voglibose (D) and the proteins 3W37, 3AJ7 and PTP1B, Table S2: Cartesian coordinates of the optimized **1** and **2** by DFT using basis M052X/6-311++G(d,p), Table S3. PTP1B and α-glucosidase inhibitory activities of total methanol extracts and its fractions from *T. ruticarpum*.

## Code Availability

(**1**) Gaussian09: EM64L—G09RevC.01—Named keywords: Density Functional Theory, BP86/def2-SVP, BP86/def2-TZVPP//BP86/def2-SVP; (**2**) MOE 2015.10 License Information—Feature: MOE; Version limit: 2016.02—Unlimited; Quickprep tool following configuration: Tether—Receptor with the strength of 5000; Refine of 0.0001 kcal∙mol^−1^∙Å^−1^, radius = 4.5 Å. Configuration: Conj Grad for minima energy; termination for energy change = 0.0001 kcal∙mol^−1^; max interactions = 1000; modify charge: Gasteiger-Huckel. Docking score energy (DS), root-mean-square deviation (RMSD), binding interactions: hydrogen bonds, cation–π, π–π bonds, ionic interactions, van der Waals interactions.

## Sample Availability

Samples of the compounds are available from the authors.

## References

[1] World Health Organization (2016). Global Report on Diabetes.

[2] Washburn W.N. (2009). Development of the Renal Glucose Reabsorption Inhibitors: A New Mechanism for the Pharmacotherapy of Diabetes Mellitus Type 2. J. Med. Chem..

[3] Moller D.E. (2001). New Drug Targets for Type 2 Diabetes and the Metabolic Syndrome. Nature.

[4] Ha M.T., Seong S.H., Nguyen T.D., Cho W.K., Ah K.J., Ma J.Y., Woo M.H., Choi J.S., Min B.S. (2018). Chalcone Derivatives from the Root Bark of *Morus alba* L. Act as Inhibitors of PTP1B and α-Glucosidase. Phytochemistry.

[5] Krishnan N., Bonham C.A., Rus I.A., Shrestha O.K., Gauss C.M., Haque A., Tocilj A., Joshua-Tor L., Tonks N.K. (2018). Harnessing Insulin-and Leptin-Induced Oxidation of PTP1B for Therapeutic Development. Nat. Commun..

[6] Johnson T.O., Ermolieff J., Jirousek M.R. (2002). Protein Tyrosine Phosphatase 1B Inhibitors for Diabetes. Nat. Rev. Drug Discov..

[7] Tomasik P., Horton D. (2012). Enzymatic Conversions of Starch.

[8] Sun Z., Henson C.A. (1990). Degradation of Native Starch Granules by Barley α-Glucosidases. Plant Physiol..

[9] Matsui H., Chiba S., Shimomura T. (1978). Substrate Specificity of an α-Glucosidase in Sugar Beet Seed. Agric. Biol. Chem..

[10] Schaechter M. (2009). Encyclopedia of Microbiology.

[11] Watanabe K., Hata Y., Kizaki H., Katsube Y., Suzuki Y. (1997). The Refined Crystal Structure of Bacillus Cereus Oligo-1,6-Glucosidase at 2.0 Å Resolution: Structural Characterization of Proline-Substitution Sites for Protein Thermostabilization. J. Mol. Biol..

[12] Hauri H.P., Quaroni A., Isselbacher K.J. (1979). Biogenesis of Intestinal Plasma Membrane: Posttranslational Route and Cleavage of Sucrase-Isomaltase. Proc. Natl. Acad. Sci. USA.

[13] Hartley T.G. (1981). Tetradium Ruticarpumo (A.Juss.).

[14] Nguyễn T.B., Nguyễn Q.B., Vũ V.C., Lê M.C., Nguyễn N.C., Vũ V.D., Nguyễn V.D., Trần Đ.D., Nguyễn K.D., Nguyễn T.D. (2000). Tên Cây Rừng Việt Nam.

[15] Li M., Wang C. (2020). Traditional Uses, Phytochemistry, Pharmacology, Pharmacokinetics and Toxicology of the Fruit of Tetradium Ruticarpum: A Review.

[16] Kato A., Yasuko H., Goto H., Hollinshead J., Nash R.J., Adachi I. (2009). Inhibitory Effect of Rhetsinine Isolated from *Evodia rutaecarpa* on Aldose Reductase Activity. Phytomedicine.

[17] Wang T., Kusudo T., Takeuchi T., Yamashita Y., Kontani Y., Okamatsu Y., Saito M., Mori N., Yamashita H. (2013). Evodiamine Inhibits Insulin-Stimulated MTOR-S6K Activation and IRS1 Serine Phosphorylation in Adipocytes and Improves Glucose Tolerance in Obese/Diabetic Mice. PLoS ONE.

[18] Tachibana K., Ishimoto K., Takahashi R., Kadono H., Awaji T., Yuzuriha T., Tanaka T., Hamakubo T., Sakai J., Kodama T. (2019). Development of a Ligand Screening Tool Using Full-Length Human Peroxisome Proliferator-Activated Receptor-Expressing Cell Lines to Ameliorate Metabolic Syndrome. Chem. Pharm. Bull..

[19] Ngo T.D., Tran T.D., Le M.T., Thai K.M. (2016). Computational Predictive Models for P-Glycoprotein Inhibition of in-House Chalcone Derivatives and Drug-Bank Compounds. Mol. Divers..

[20] Chandra Babu T.M., Rajesh S.S., Bhaskar B.V., Devi S., Rammohan A., Sivaraman T., Rajendra W. (2017). Molecular Docking, Molecular Dynamics Simulation, Biological Evaluation and 2D QSAR Analysis of Flavonoids from Syzygium Alternifolium as Potent Anti-Helicobacter Pylori Agents. RSC Adv..

[21] Thai K.M., Le D.P., Tran N.V.K., Nguyen T.T.H., Tran T.D., Le M.T. (2015). Computational Assay of Zanamivir Binding Affinity with Original and Mutant Influenza Neuraminidase 9 Using Molecular Docking. J. Theor. Biol..

[22] Tarasova O., Poroikov V., Veselovsky A. (2018). Molecular Docking Studies of HIV-1 Resistance to Reverse Transcriptase Inhibitors: Mini-Review. Molecules.

[23] Ding Y., Fang Y., Moreno J., Ramanujam J., Jarrell M., Brylinski M. (2016). Assessing the Similarity of Ligand Binding Conformations with the Contact Mode Score. Comput. Biol. Chem..

[24] Zhao N., Li Z.L., Li D.H., Sun Y.T., Shan D.T., Bai J., Pei Y.H., Jing Y.K., Hua H.M. (2015). Quinolone and Indole Alkaloids from the Fruits of *Euodia rutaecarpa* and Their Cytotoxicity against Two Human Cancer Cell Lines. Phytochemistry.

[25] Liu Z.L., Chu S.S., Jiang G.H. (2009). Feeding Deterrents from *Zanthoxylum schinifolium* against Two Stored-Product Insects. J. Agric. Food Chem..

[26] Nguyen P.H., Yang J.L., Uddin M.N., Park S.L., Lim S.I., Jung D.W., Williams D.R., Oh W.K. (2013). Protein Tyrosine Phosphatase 1B (PTP1B) Inhibitors from *Morinda citrifolia* (Noni) and Their Insulin Mimetic Activity. J. Nat. Prod..

[27] Lu L., Li Z.H., Shan C.Y., Ma S.H., Nie W., Wang H.B., Chen G.Q., Li S.H., Shu C.J. (2021). Whole transcriptome analysis of schinifoline treatment in *Caenorhabditis elegans* infected with *Candida albicans*. Mol. Immunol..

[28] Nguyen P.H., Zhao B.T., Kim K.W., Lee J.H., Choi J.S., Min B.S., Woo M.H. (2016). Anti-inflammatory terpenylated coumarins from the leaves of *Zanthoxylum schinifolium* with a-glucosidase inhibitory activity. J. Nat. Med..

[29] Wang C.F., Fan L., Tian M., Qi X.S., Liu J.X., Feng J.B., Du S.S., Su X., Wang Y.Y. (2014). Radiosensitizing Effect of Schinifoline from *Zanthoxylum schinifolium* Sieb et Zucc on Human Non-Small Cell Lung Cancer A549 Cells: A Preliminary in Vitro Investigation. Molecules.

[30] Wang X.X., Zan K., Shi S.P., Zeng K.W., Jiang Y., Guan Y., Xiao C.L., Gao H.Y., Wu L.J., Tu P.F. (2013). Quinolone alkaloids with antibacterial and cytotoxic activities from the fruits of *Evodia rutaecarpa*. Fitoterapia.

[31] Fang Z., Jun D.Y., Kim Y.H., Min B.S., Kim A.K., Woo M.H. (2010). Cytotoxic Constituents from the Leaves of *Zanthoxylum schinifolium*. Bull. Korean Chem. Soc..

[32] Bai J.W., Zhang Y., Yang M.J. (2000). Effects of schinifoline on cytoskeleton of experimental hepatoma in rats observed by whole mount cell transmission electron microscopy. Beijing Zhongyiyao Daxue Xuebao.

[33] Bai J.W., Zhang Y., Wu J. (1999). Flow cytometric analysis of the schinifoline inhibition on rat hepatoma cell induced by DEN. Beijing Zhongyiyao Daxue Xuebao.

[34] Thomas G. (2011). Medicinal Chemistry: An Introduction.

[35] Sessler J.L., Gale P.A., Cho W.-S. (2006). Anion Receptor Chemistry.

[36] Rice-Evans C.A., Miller N.J., Paganga G. (1996). Structure-antioxidant activity relationships of flavonoids and phenolic acids. Free Radic. Biol. Med..

[37] Carvalho A.A., Andrade L.N., Batista É., Sousa V., De Sousa D.P. (2015). Antitumor Phenylpropanoids Found in Essential Oils. BioMed Res. Int..

[38] Bezerra D.P., De Morais M.C. (2017). The Dual Antioxidant/Prooxidant Effect of Eugenol and Its Action in Cancer Development and Treatment. Nutrients.

[39] Kharkyanen V.N., Petrov E.G., Ukrainskii I.I. (1978). Donor-Acceptor Model of Electron Transfer through Proteins. J. Theor. Biol..

[40] Bui T.Q., Phuong Loan H.T., Ai My T.T., Quang D.T., Phuong Thuy B.T., Nhan V.D., Quy P.T., Van Tat P., Dao D.Q., Trung N.T. (2020). A Density Functional Theory Study on Silver and Bis-Silver Complexes with Lighter Tetrylene: Are Silver and Bis-Silver Carbenes Candidates for SARS-CoV-2 Inhibition Insight from Molecular Docking Simulation. RSC Adv..

[41] Frisch H.B.M.J., Schlegel G.E., Scuseria M.A., Robb J.R., Cheeseman G., Scalmani V., Barone B., Mennucci G.A., Petersson H., Nakatsuji M. (2009). Gaussian 09.

[42] Markovi Z.S., Dimitri J.M. (2011). Mechanistic Study of the Structure—Activity Relationship for the Free Radical Scavenging Activity of Baicalein. J. Mol. Model..

[43] Weigend F., Ahlrichs R. (2005). Balanced Basis Sets of Split Valence, Triple Zeta Valence and Quadruple Zeta Valence Quality for H to Rn: Design and Assessment of Accuracy. Phys. Chem. Chem. Phys..

[44] Reed A.E., Weinstock R.B., Weinhold F. (1985). Natural Population Analysis. J. Chem. Phys..

[45] Koopmans T. (1934). Über Die Zuordnung von Wellenfunktionen Und Eigenwerten Zu Den Einzelnen Elektronen Eines Atoms. Physica.

[46] De Chimie L., Theorique P. (1999). Chemical Reactivity Indexes in Density. J. Comput. Chem..

[47] Obot I.B., Macdonald D.D., Gasem Z.M. (2015). Density Functional Theory (DFT) as a Powerful Tool for Designing New Organic Corrosion Inhibitors. Corros. Sci..

