# Supplementary material for: On the Inhibitability of Natural Products Isolated from Tetradium ruticarpum towards Tyrosine Phosphatase 1B (PTP1B) and α-Glucosidase (3W37): An In Vitro and In Silico Study
Source: Molecules. 2021 Jun 17;26(12):3691. doi: 10.3390/molecules26123691 (PMC8233831; doi:10.3390/molecules26123691)
Supplement: Supplementary file 1 [file molecules-26-03691-s001.zip › molecules-1267168-supplementary.pdf]

Supplementary data

On the Inhibitability of Natural Products Isolated from *Tetradium ruticarpum* towards Tyrosine Phosphatase 1B (PTP1B) and α-glucosidase (3W37): an *in vitro* and *in silico* Study

Dao-Cuong To[1,2§], Thanh Q. Bui[3,§], Nguyen Thi Ai Nhung[3], Quoc Toan Tran[4], Thi-Thuy Do[4], Manh-Hung Tran[5], Phan Phuoc Hien[6], Phan Tu Quy[7], The-Hung Nguyen[8], Huu-Tho Nguyen[8], Tien-Dung Nguyen[8,9]*, Phi-Hung Nguyen[4]*

[1] *Phenikaa University, Yen Nghia, Ha Dong district, Hanoi 12116, Vietnam*

[2]*Phenikaa Research and Technology Institute (PRATI), A&A Green Phoenix Group JSC, 167 Hoang Ngan, Cau Giay District, Hanoi 11313, Vietnam*

[3]*Department of Chemistry, University of Sciences, Hue University, Hue City 530000, Vietnam*

[4] *Institute of Natural Products Chemistry, Vietnam Academy of Science and Technology (VAST), 18 Hoang Quoc Viet, Cau Giay district, Hanoi 122100, Vietnam*

[5]*Faculty of Pharmacy, Dong A University, Da Nang City 550000, Vietnam*

[6]*Institute of Applied Science and Technology, Van Lang University, Ho Chi Minh City, 700000 Vietnam*

[7]*Department of Natural Sciences & Technology, Tay Nguyen University, Buon Ma Thuot, Dak Lak 630000 Vietnam*

[8] *College of Agriculture and Forestry, Thai Nguyen University (TUAF), Quyet Thang 24119, Vietnam*

[9] *Institute of Forestry Research and Development, TUAF, Quyet Thang 24119, Vietnam*

[§]These authors equally contributed to this work.

*Correspondence to:

Phi-Hung Nguyen (E-mail: nguyenphihung1002@gmail.com)

Tien-Dung Nguyen (E-mail: dungnt@tuaf.edu.vn)

Contents

1. Experimental results

Figure S1. ^{1}H NMR spectrum of compound **1** (400 MHz, $CDCl_3$)

Figure S2. ^{1}H NMR spectrum of compound **1** (400 MHz, $CDCl_3$) (expanded)

Figure S3. ^{1}H NMR spectrum of compound **1** (400 MHz, $CDCl_3$) (expanded)

Figure S4. ^{13}C NMR spectrum of compound **1** (100 MHz, $CDCl_3$)

Figure S5. ^{13}C NMR spectrum of compound **1** (100 MHz, $CDCl_3$) (expanded)

Figure S6. HMQC spectrum of compound **1** ($CDCl_3$)

Figure S7. HMBC spectrum of compound **1** ($CDCl_3$

Figure S8. Mass spectrum of compound **1**

Figure S9. ^{1}H NMR spectrum of compound **2** (400 MHz, $CDCl_3$)

Figure S10. ^{13}C NMR spectrum of compound **2** (100 MHz, $CDCl_3$)

Figure S11. DEPT spectrum of compound **2** ($CDCl_3$)

Figure S12. COSY spectrum of compound **2** ($CDCl_3$)

Figure S13. HSQC spectrum of compound **2** ($CDCl_3$)

Figure S14. HMBC spectrum of compound **2** ($CDCl_3$

Figure S15. Mass spectrum of compound **2**

2. Computational results

Table S1. Molecular docking simulation results for inhibitory complexes between the controlled drug voglibose (**D**) and the proteins 3W37, 3AJ7 and PTP1B

Figure S16. Visual presentation and in-pose interaction map of voglibose-protein inhibitory complexes: (a) **D-3W37**, (b) **D-3AJ7**, (c) **D-PTP1B**

Table S2. Cartesian coordinates of the optimized **1** and **2** by DFT using basis M052X/6-311++G(d,p)

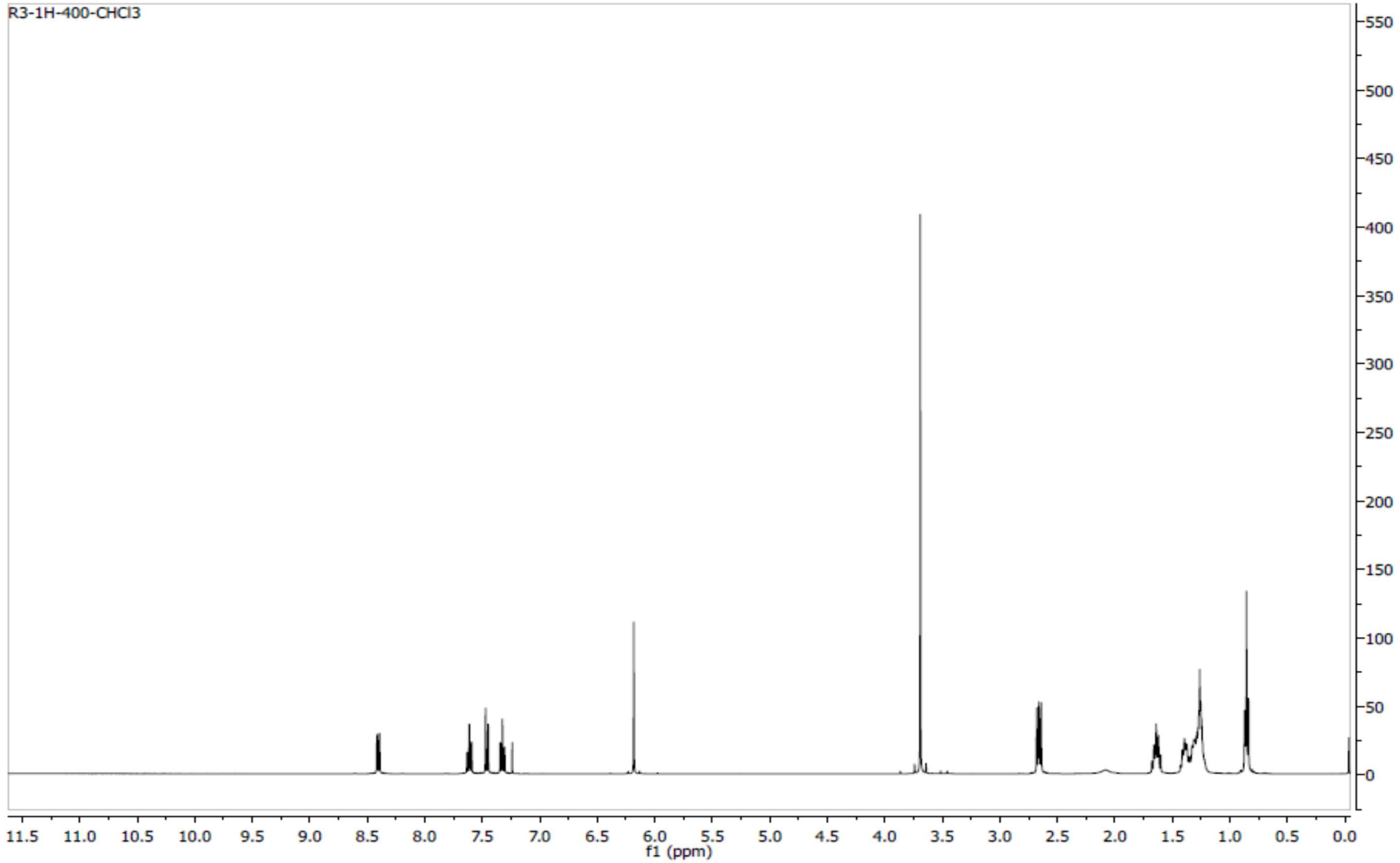

Figure S1. ^{1}H NMR spectrum of compound **1** (400 MHz, $CDCl_3$)

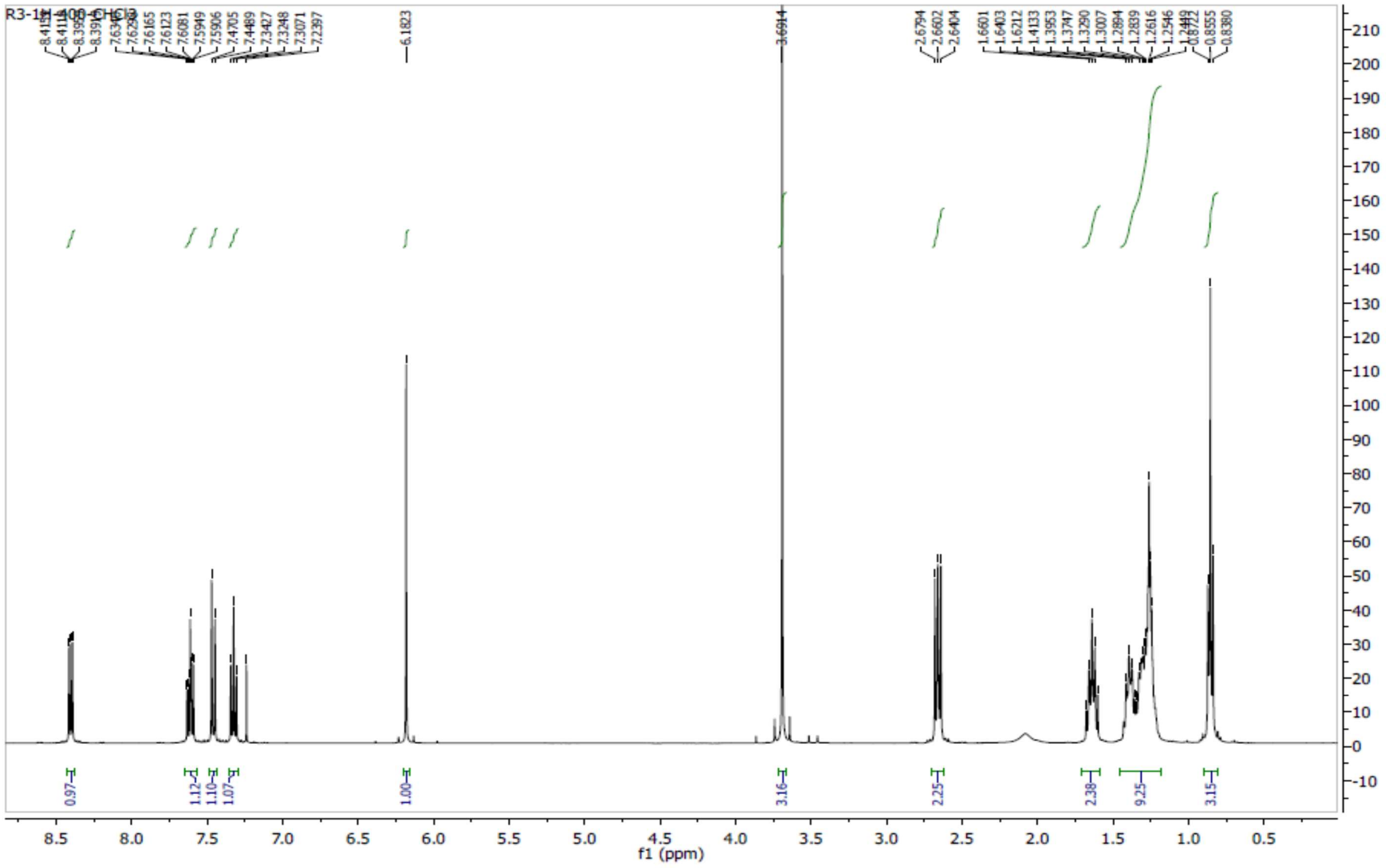

Figure S2. ^{1}H NMR spectrum of compound **1** (400 MHz, $CDCl_3$) (expanded)

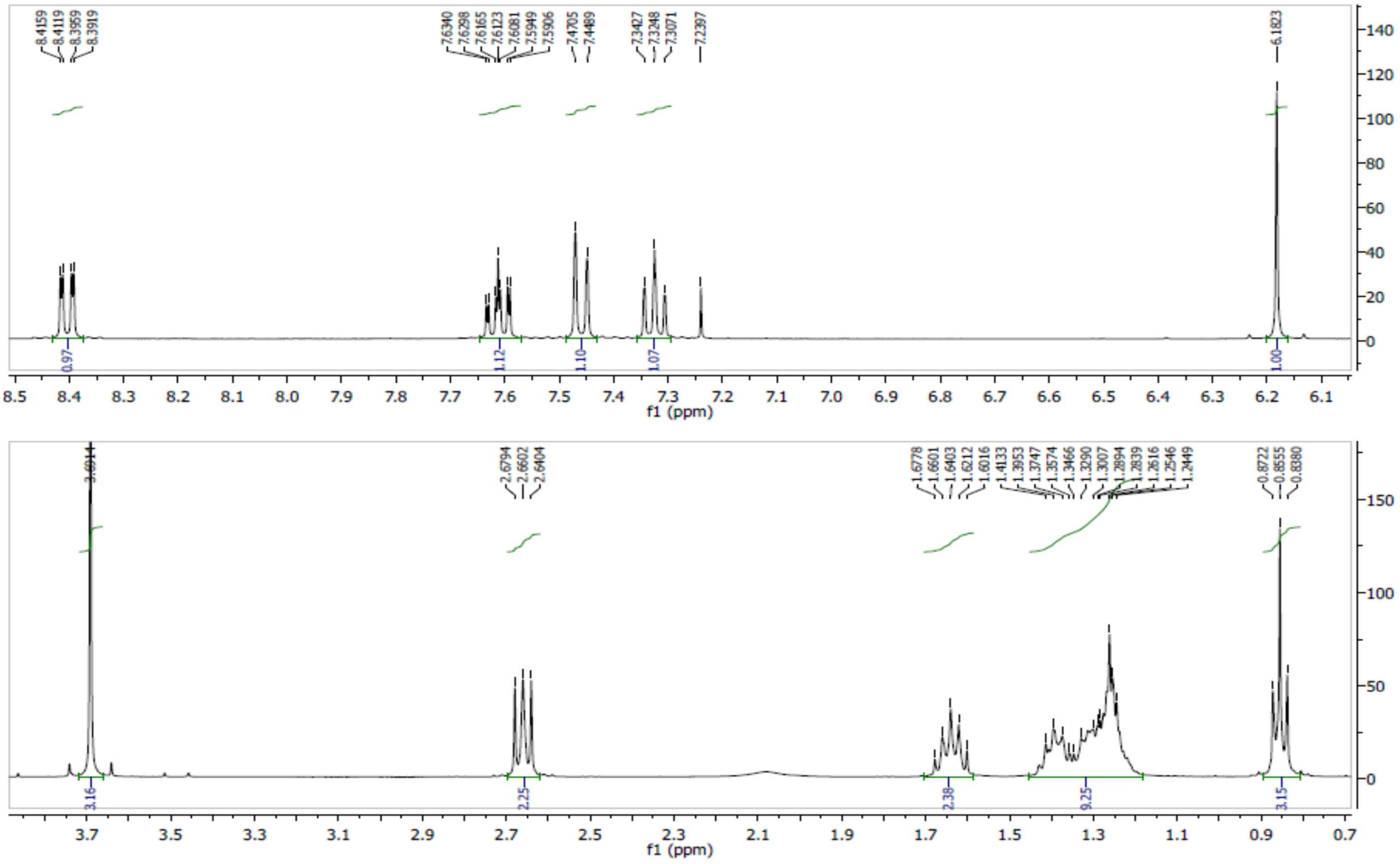

Figure S3. ^{1}H NMR spectrum of compound **1** (500 MHz, $CDCl_3$) (expanded)

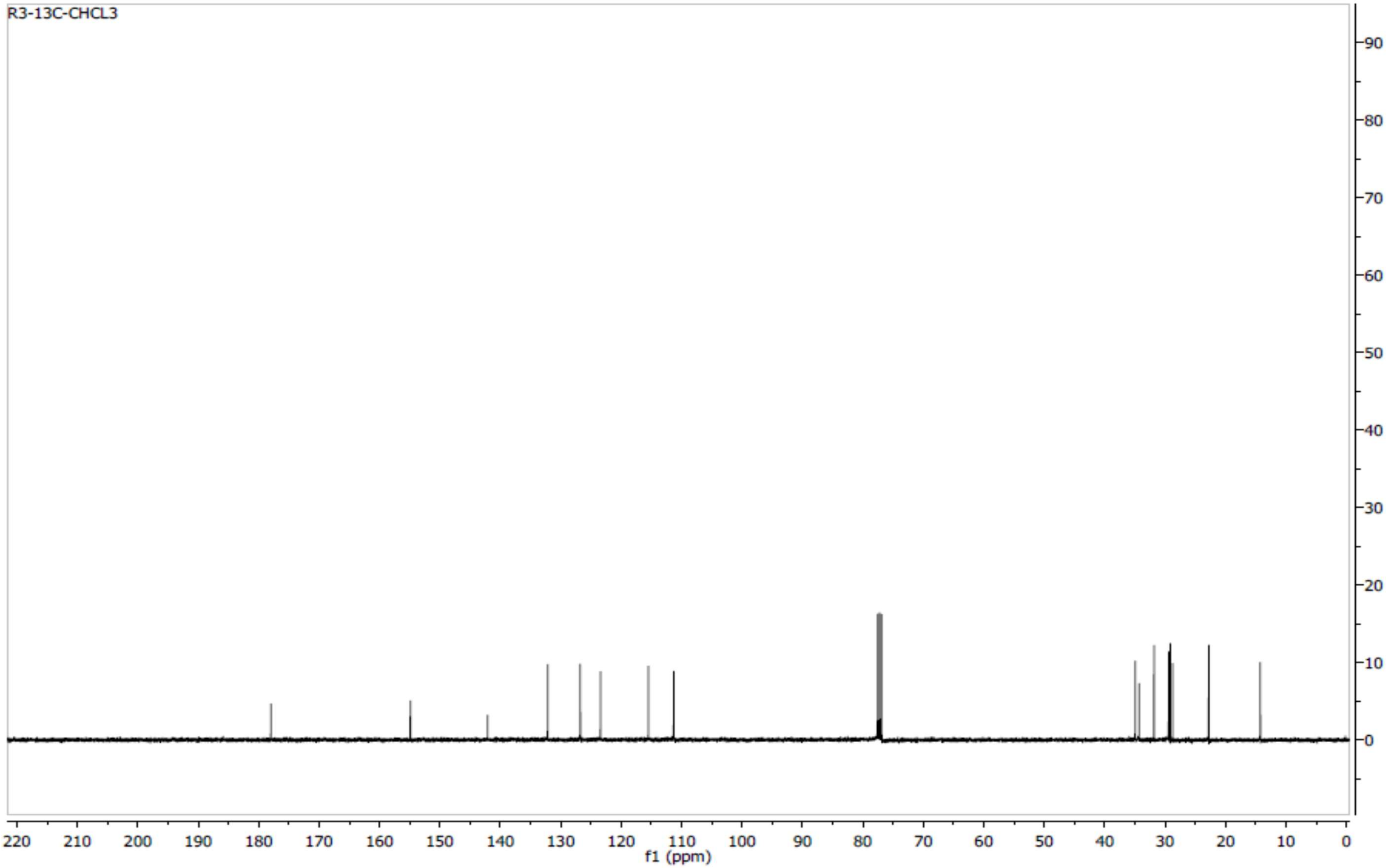

Figure S4. ^{13}C NMR spectrum of compound **1** (100 MHz, $CDCl_3$)

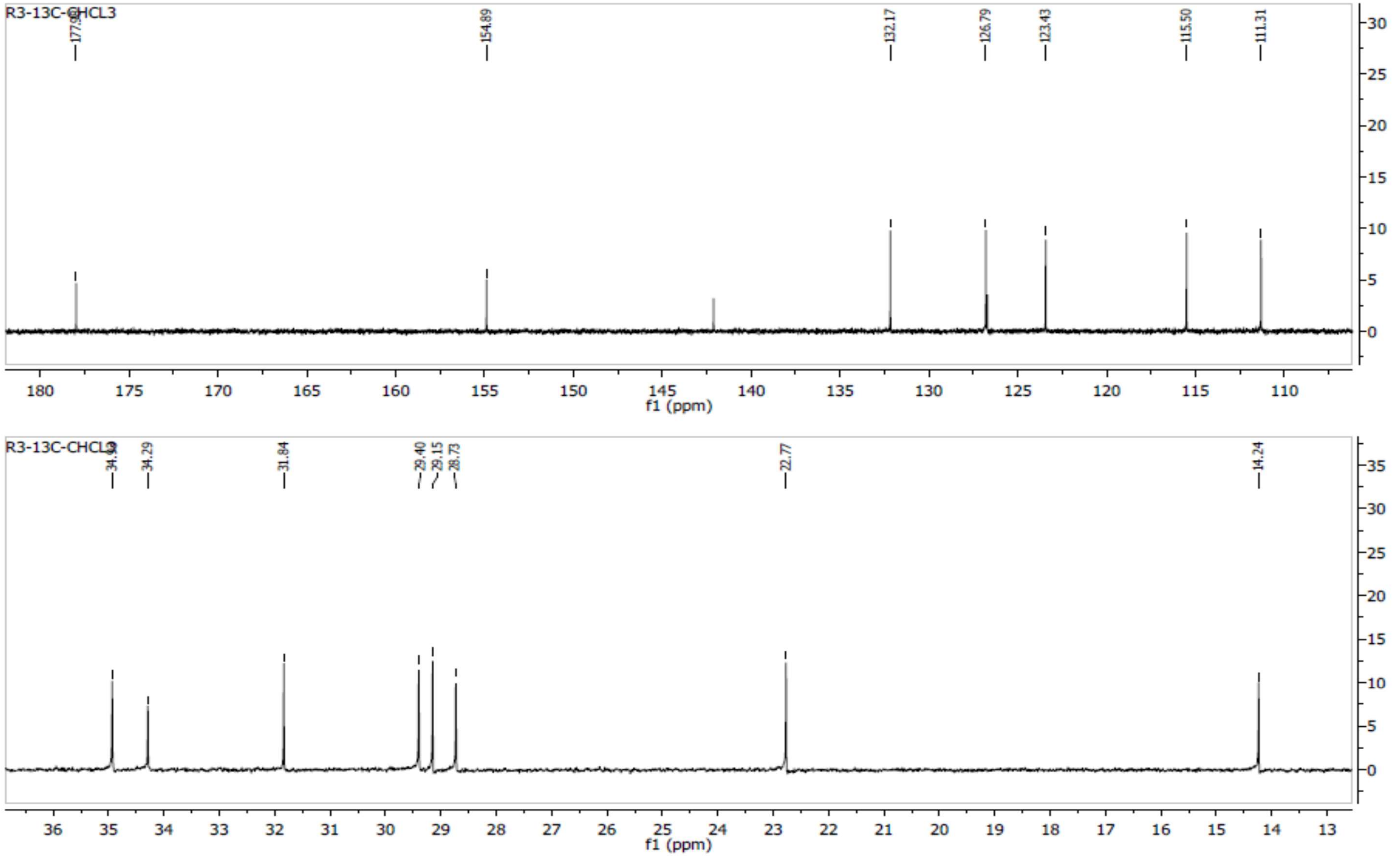

Figure S5. ^{13}C NMR spectrum of compound **1** (125 MHz, $CDCl_3$) (expanded)

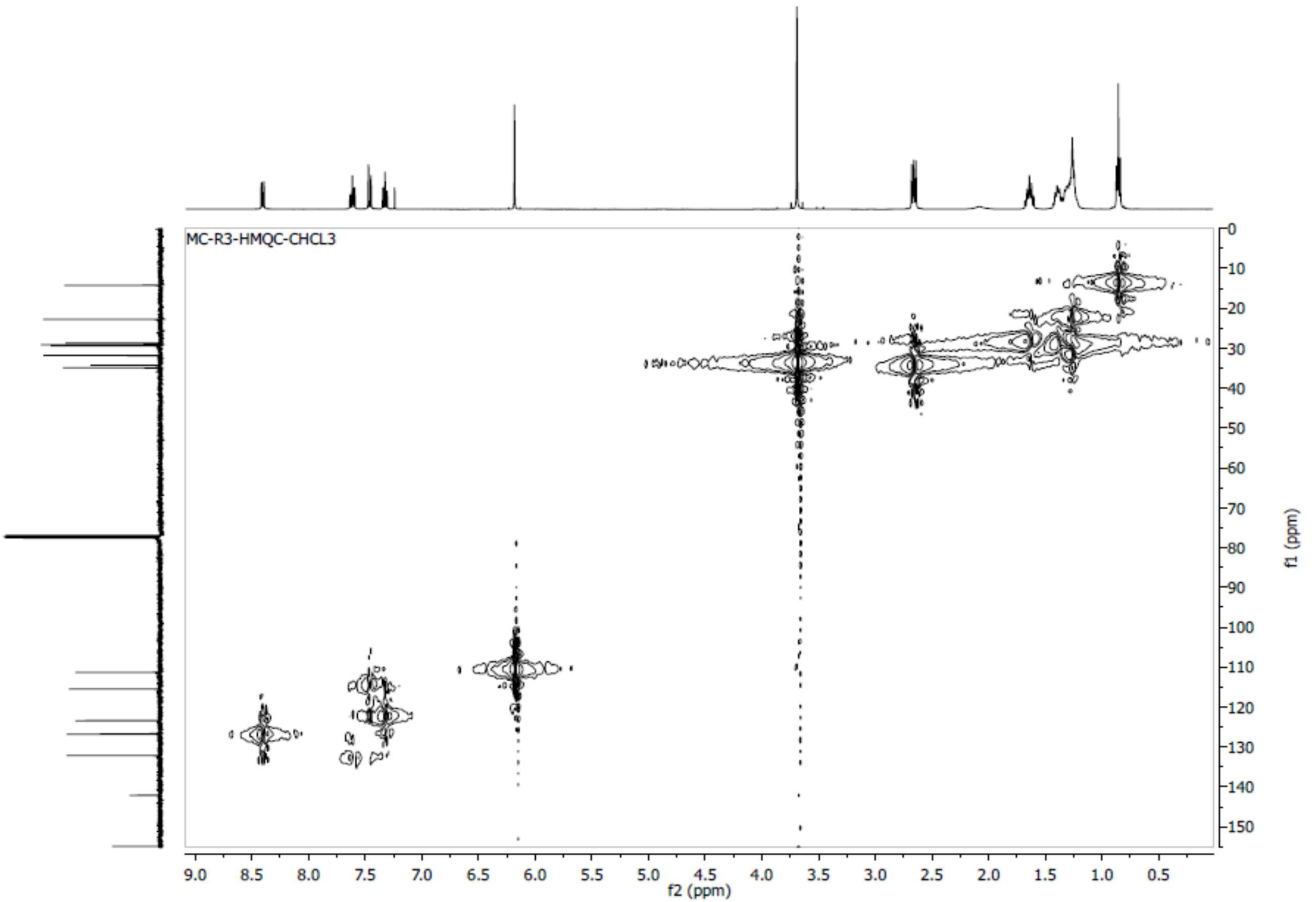

Figure S6. HMQC spectrum of compound **1**

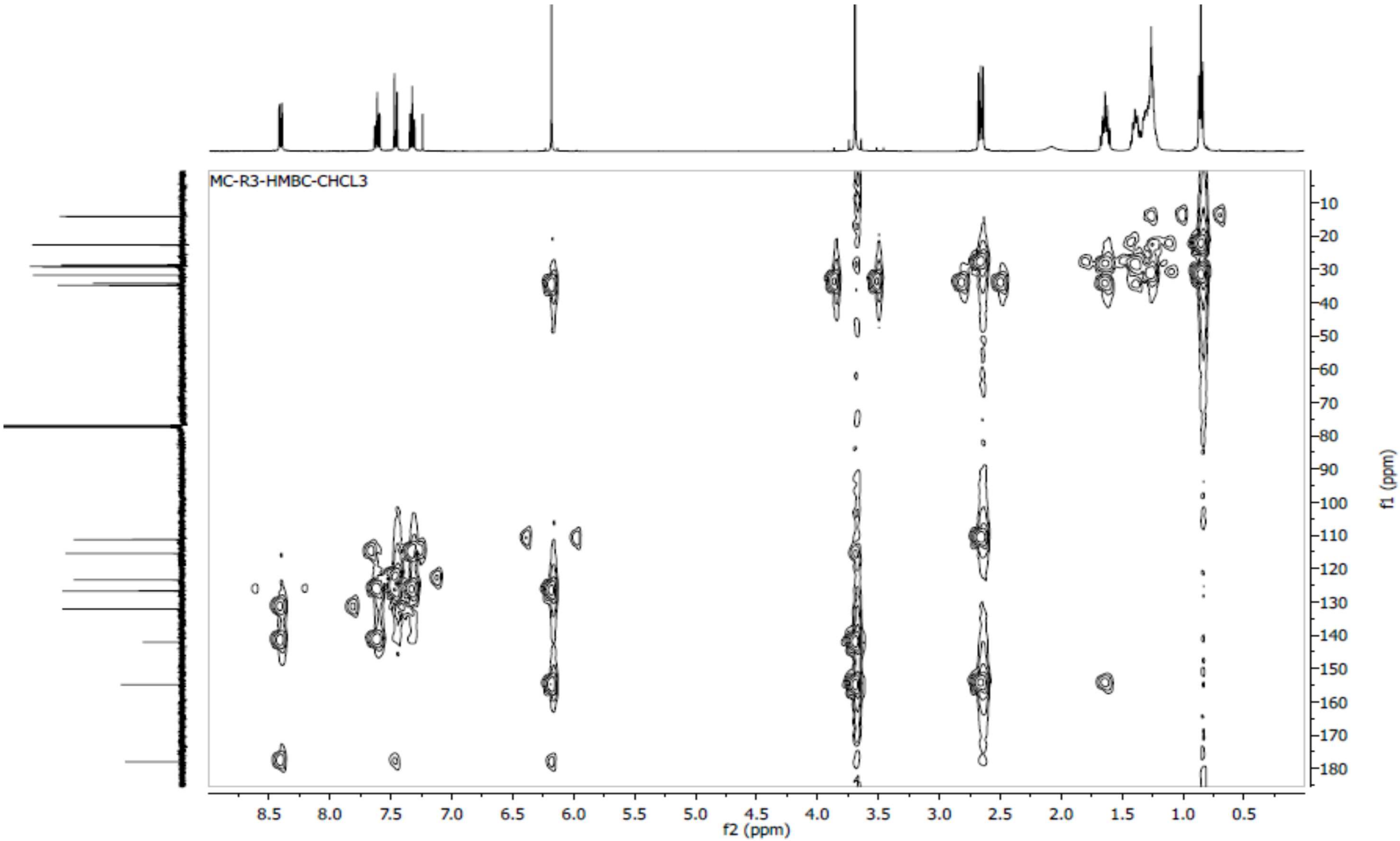

Figure S7. HMBC spectrum of compound **1**

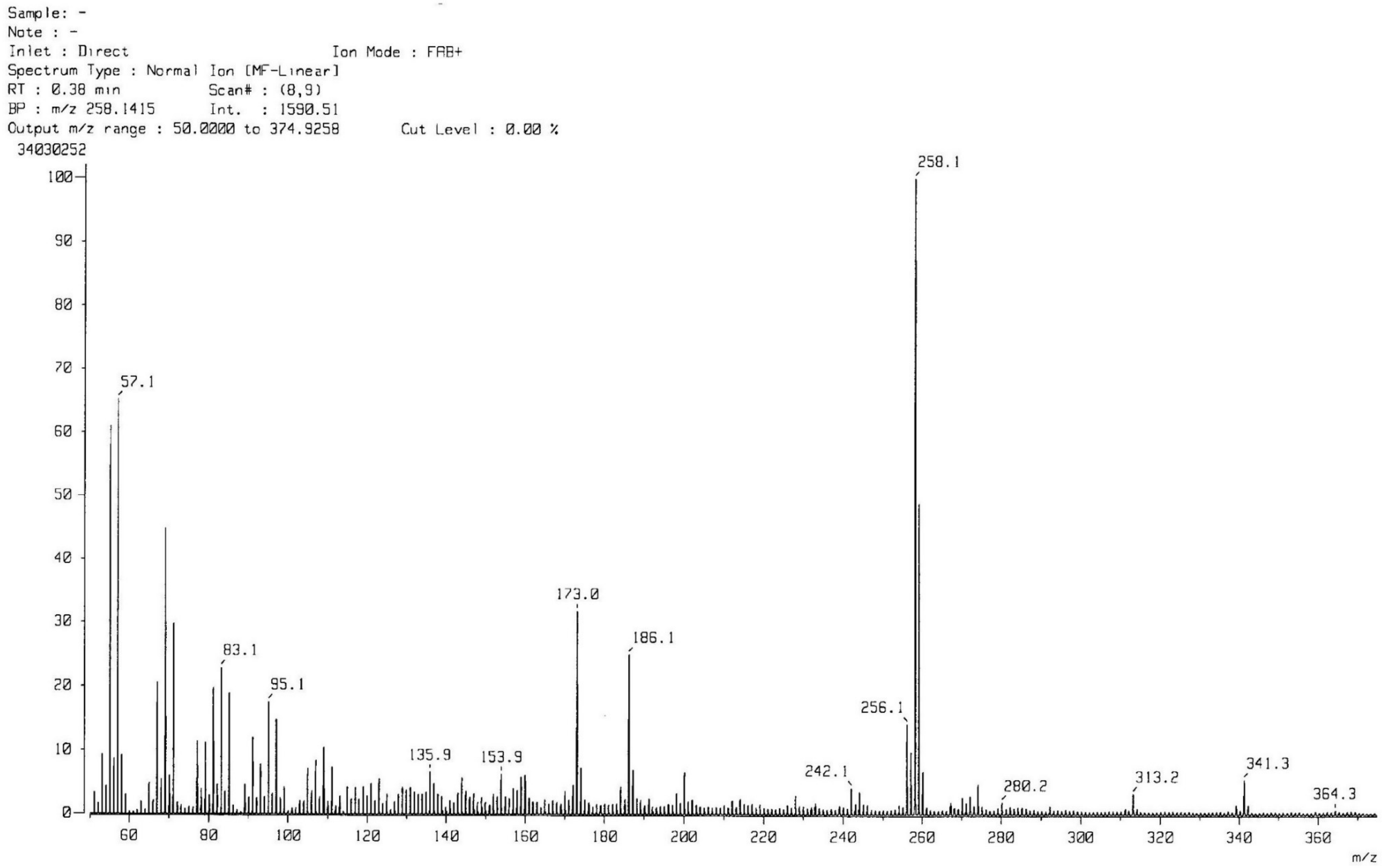

Figure S8. Mass spectrum of compound **1**

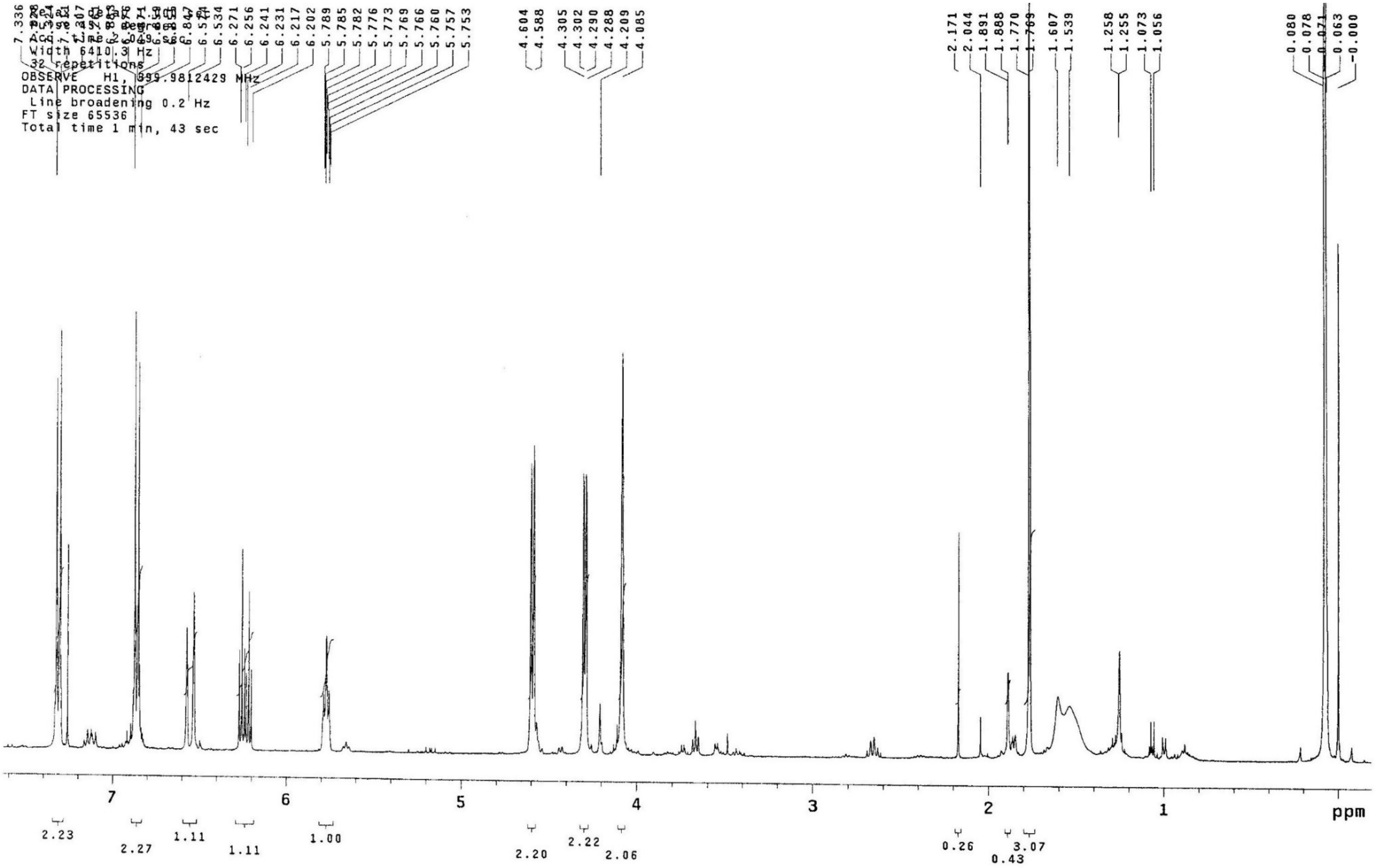

Figure S9. ^{1}H NMR spectrum of compound **2** (400 MHz, $CDCl_3$)

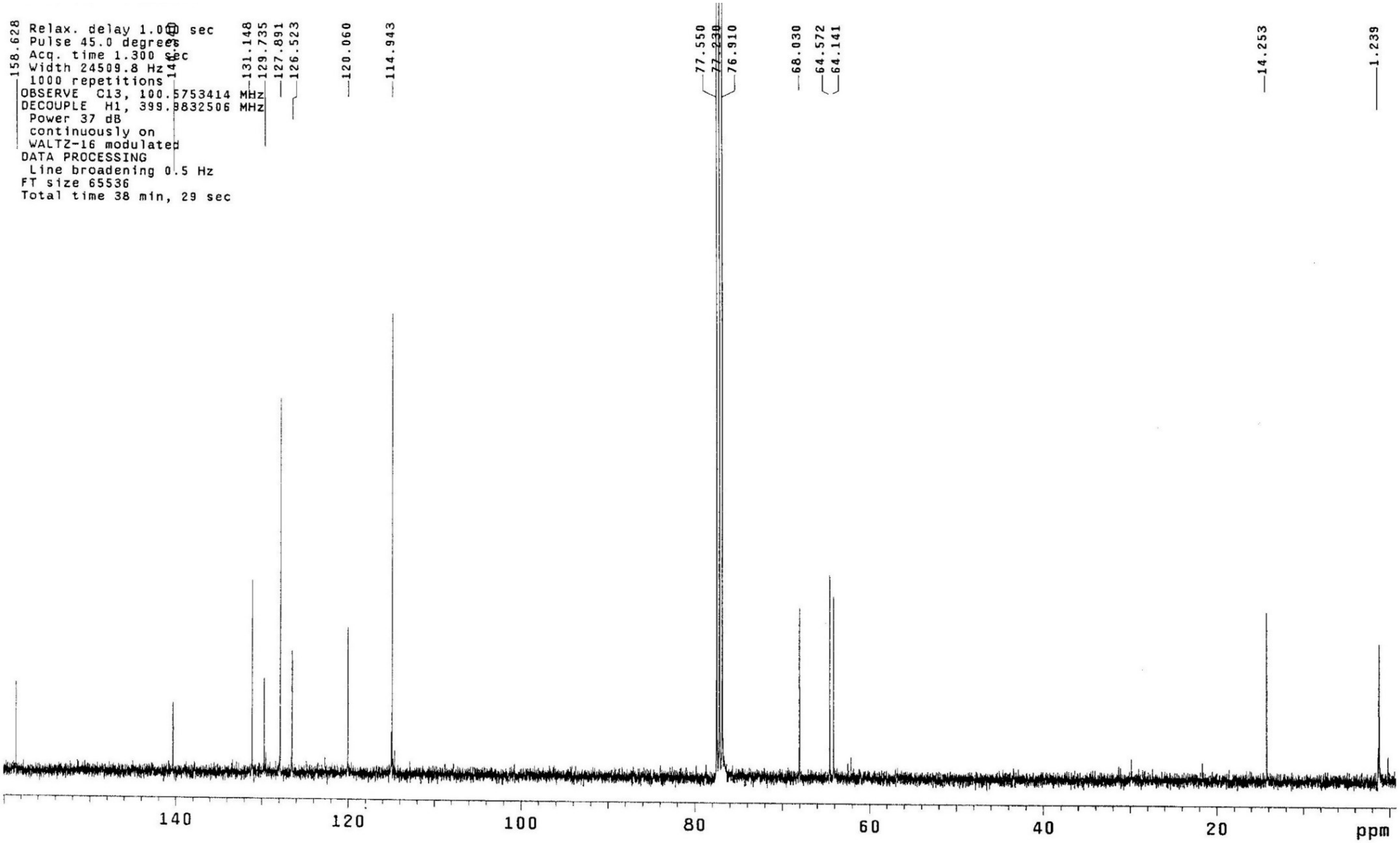

Figure S10. ^{13}C NMR spectrum of compound **2** (100 MHz, $CDCl_3$)

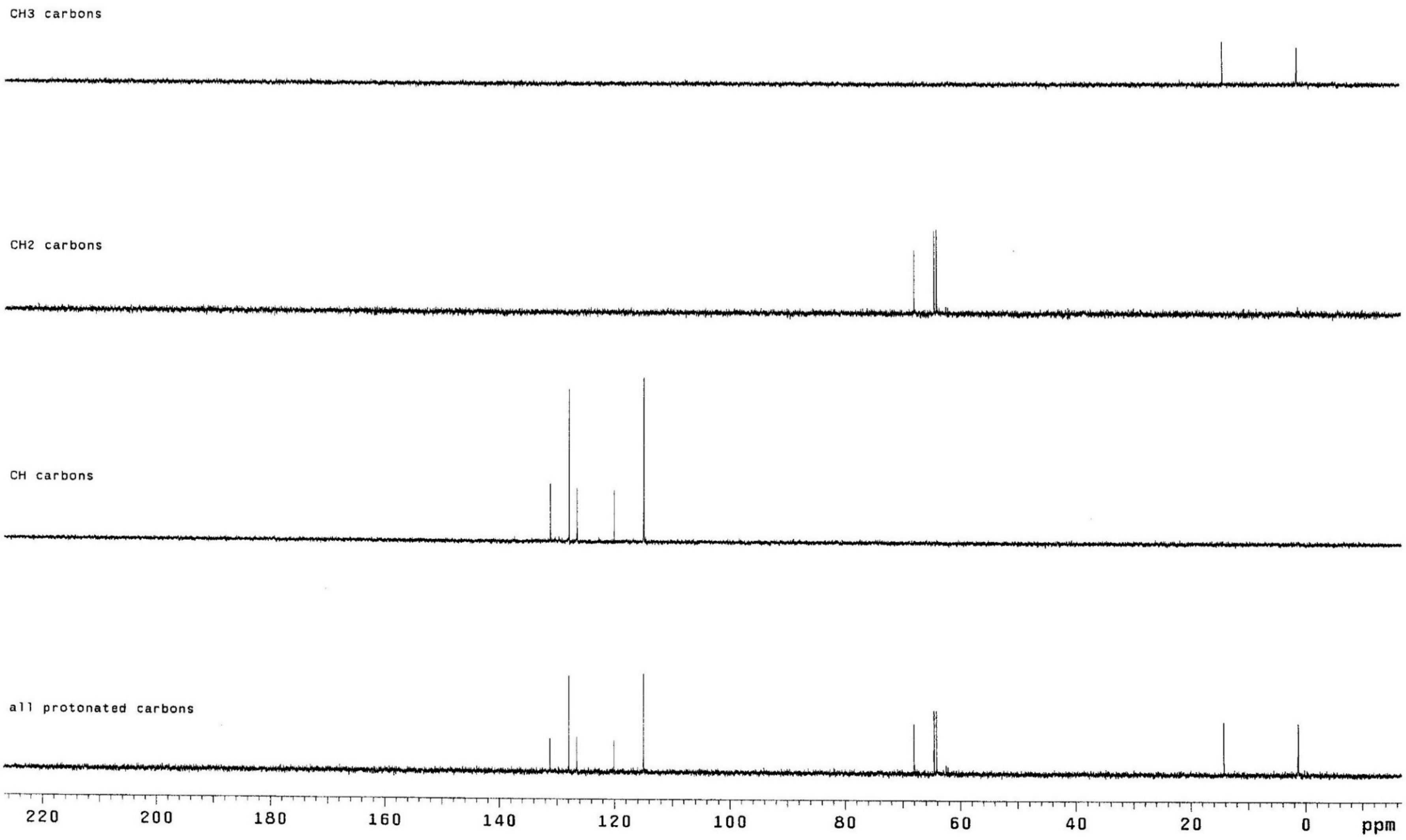

Figure S11. DEPT spectrum of compound **2**

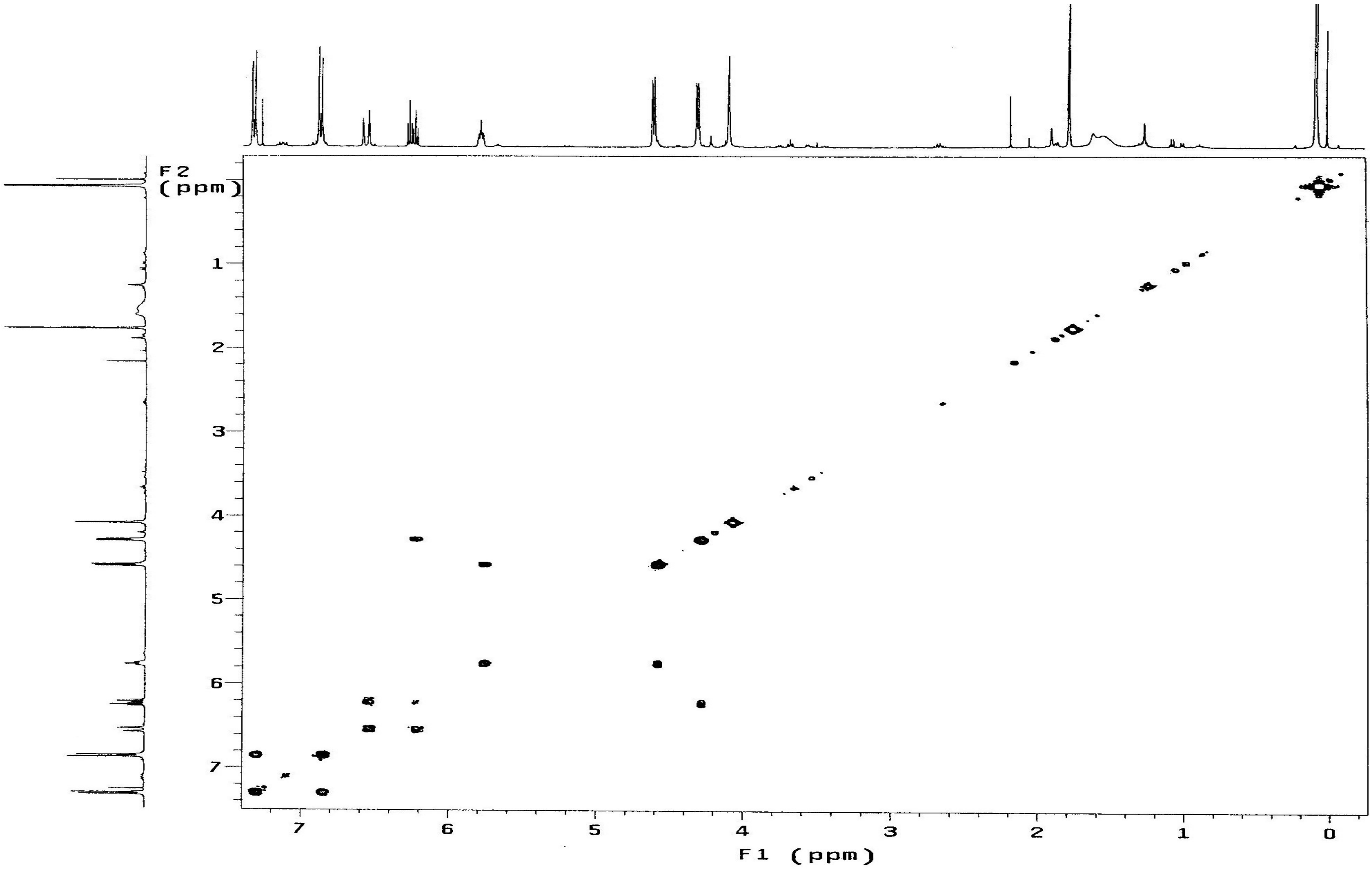

Figure S12. COSY spectrum of compound **2**

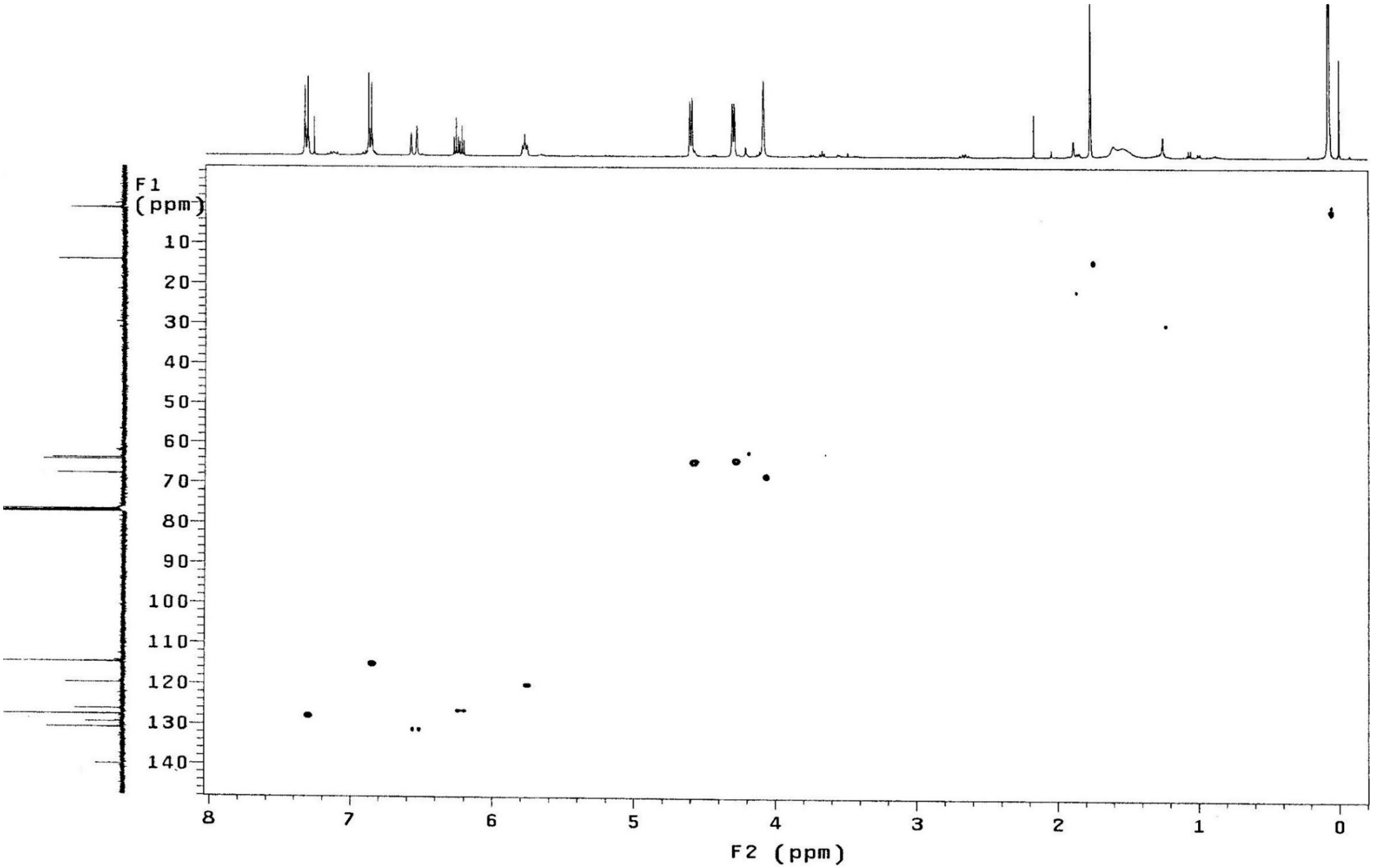

Figure S13. HSQC spectrum of compound **2**

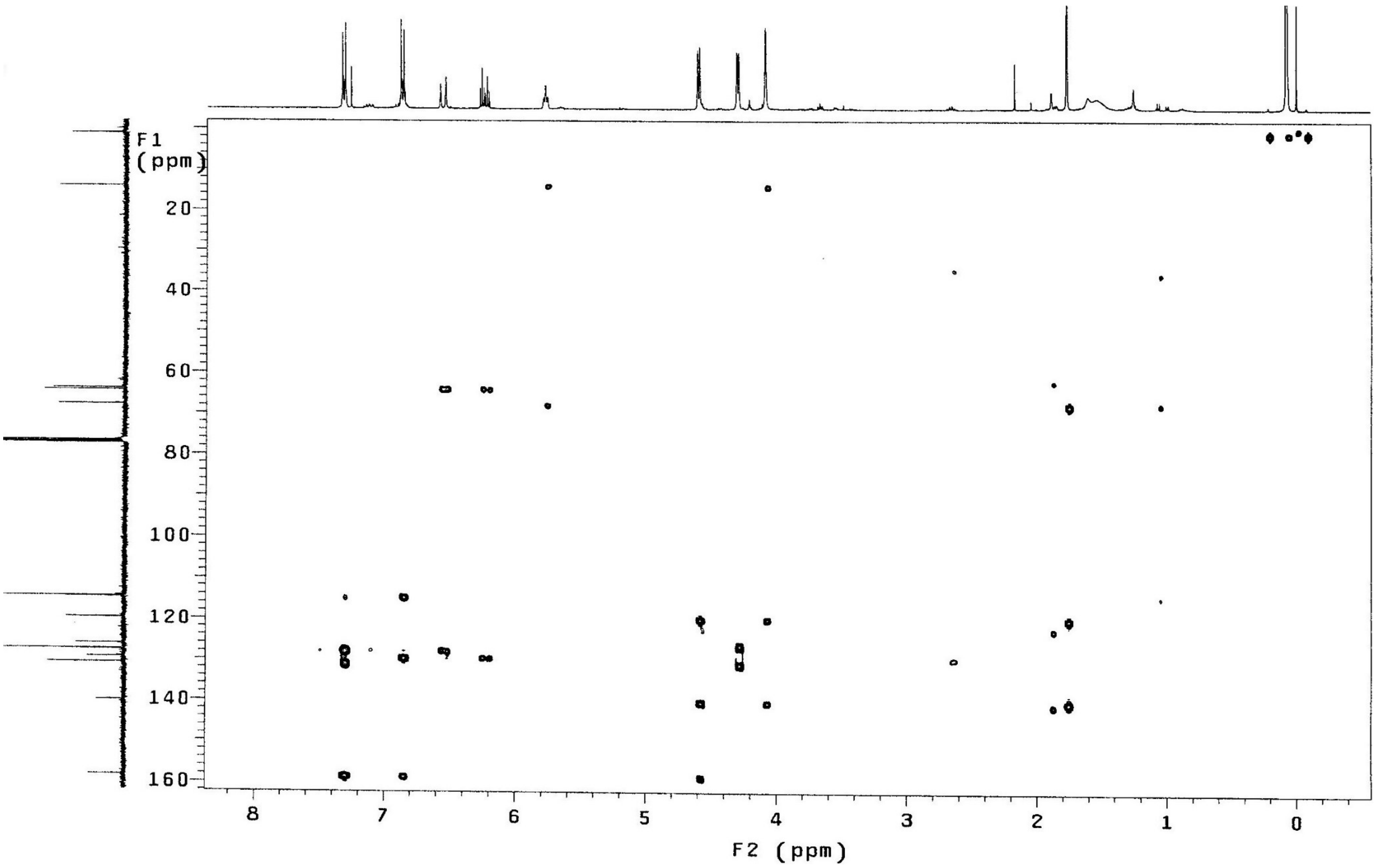

Figure S14. HMBC spectrum of compound **2**

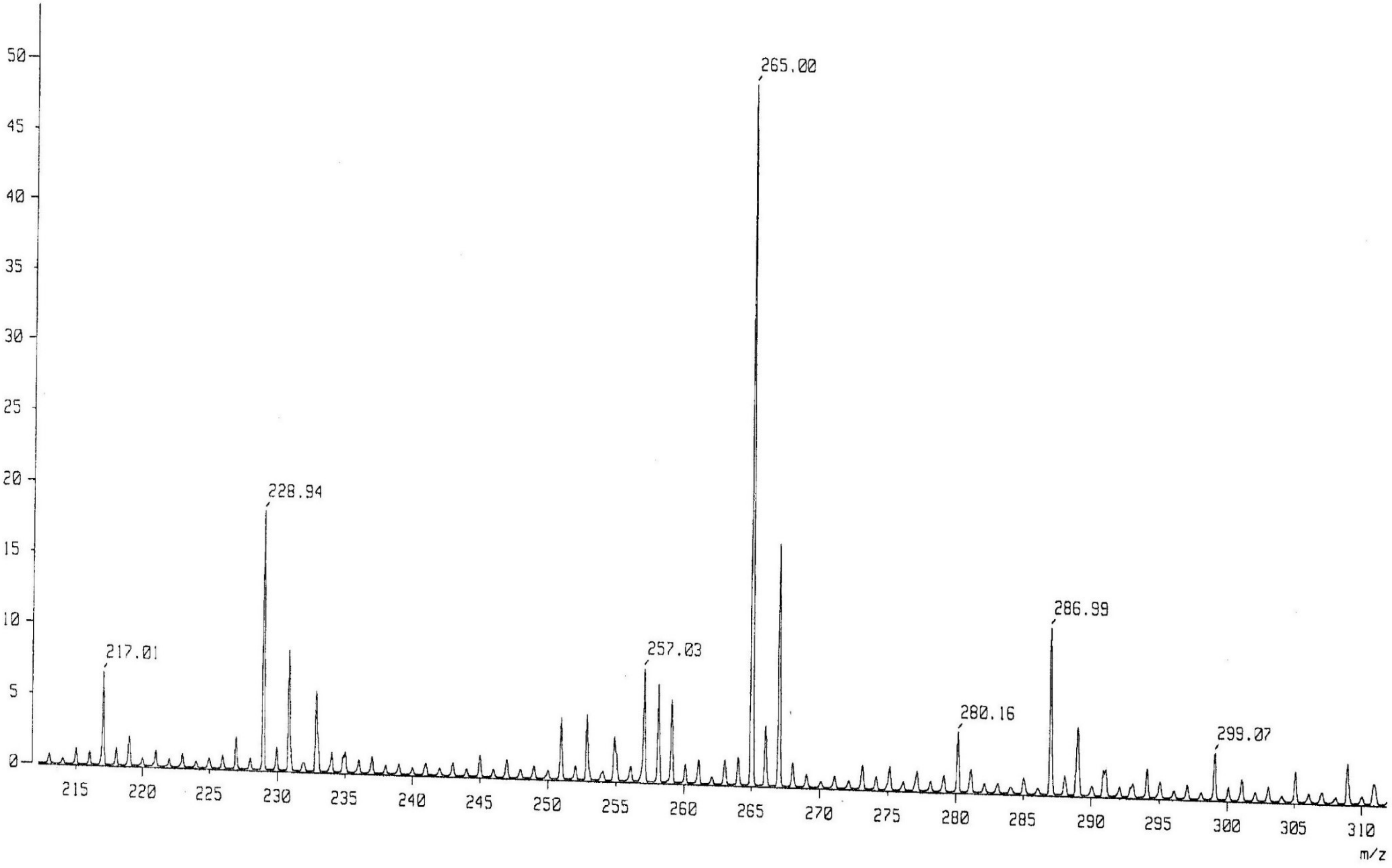

Figure S15. Mass spectrum of compound **2**

Table S1. Molecular docking simulation results for inhibitory complexes between the controlled drug voglibose (**D**) and the proteins 3W37, 3AJ7 and PTP1B

<table>
<tr><th colspan="3">Ligand-protein complex</th><th colspan="6">Hydrogen bond</th><th rowspan="2">van der Waals interaction</th></tr>
<tr><th>Name</th><th>DS</th><th>RMSD</th><th>L</th><th colspan="2">P</th><th>T</th><th>D</th><th>E</th></tr>
<tr><td rowspan="4">D-3W37</td><td rowspan="4">-14.1</td><td rowspan="4">1.77</td><td>O</td><td>O</td><td>Glu792</td><td>H-donor</td><td>2.94</td><td>-1.2</td><td rowspan="4">Thr662, Asp666, Leu663, Gly791, Tyr659, Val760, Thr790, Gly698, Asn758, Arg699, Arg676, Ile671</td></tr>
<tr><td>O</td><td>O</td><td>Ile759</td><td>H-donor</td><td>2.79</td><td>-1.4</td></tr>
<tr><td>O</td><td>O</td><td>Glu792</td><td>H-donor</td><td>2.88</td><td>-1.6</td></tr>
<tr><td>O</td><td>O</td><td>Arg670</td><td>H-acceptor</td><td>2.91</td><td>-0.7</td></tr>
<tr><td rowspan="4">D-3AJ7</td><td rowspan="4">-13.6</td><td rowspan="4">1.02</td><td>O</td><td>O</td><td>Asp352</td><td>H-donor</td><td>2.78</td><td>-1.1</td><td rowspan="4">Phe303, Gln279, Thr306, Phe159, Tyr158, Phe178, Glu277, Asn350, Val216, Arg315, Asp215</td></tr>
<tr><td>O</td><td>O</td><td>Glu411</td><td>H-donor</td><td>3.15</td><td>-1.5</td></tr>
<tr><td>O</td><td>N</td><td>Arg4429</td><td>H-acceptor</td><td>3.07</td><td>-1.4</td></tr>
<tr><td>O</td><td>N</td><td>Gln353</td><td>H-acceptor</td><td>2.84</td><td>-1.5</td></tr>
<tr><td rowspan="4">D-PTP1B</td><td rowspan="4">-14.5</td><td rowspan="4">1.40</td><td>O</td><td>O</td><td>Lys41</td><td>H-donor</td><td>3.00</td><td>-1.1</td><td rowspan="4">Asn44. Ala35, Arg45, Tyr46, Lys36, Ser50</td></tr>
<tr><td>O</td><td>C</td><td>Lys41</td><td>H-acceptor</td><td>3.31</td><td>-0.9</td></tr>
<tr><td>O</td><td>N</td><td>Lys41</td><td>H-acceptor</td><td>3.17</td><td>-0.8</td></tr>
<tr><td>N</td><td>C</td><td>Arg47</td><td>H-acceptor</td><td>3.39</td><td>-0.8</td></tr>
<tr><td colspan="10">DS: Docking score energy (kcal.mol^{-1}); RMSD: Root-mean-square deviation (Å)</td></tr>
<tr><td colspan="10">L: Ligand; P: Protein; T: Type; D: Distance (Å); E: Energy (kcal.mol^{-1})</td></tr>
</table>

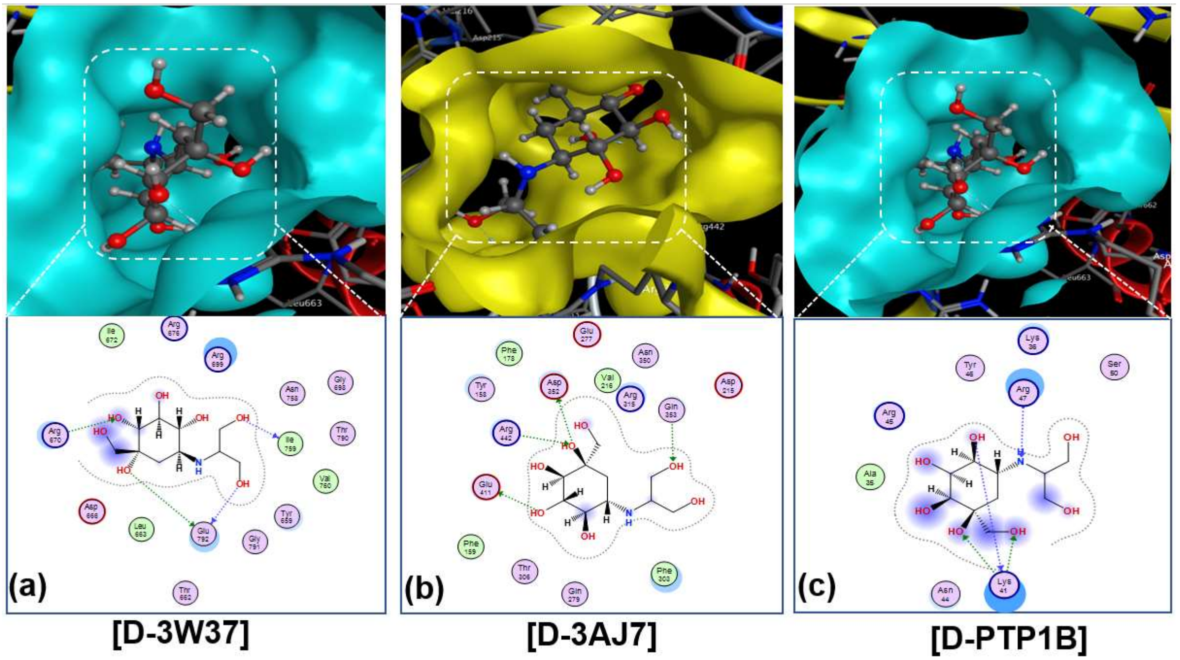

Figure S16. Visual presentation and in-pose interaction map of voglibose-protein inhibitory complexes: (a) **D-3W37**, (b) **D-3AJ7**, (c) **D-PTP1B**.

Table S2. Cartesian coordinates of the optimized **1** and **2** by DFT using basis M052X/6-311++G(d,p)

2-heptyl-1-methylquinolin-4-one (**1**)

C	5.75751200	0.41462600	0.00470900
H	6.76740300	0.79843300	0.00644600
C	5.52590100	-0.96210500	0.00402200
H	6.36091300	-1.64945800	0.00523600
C	4.24277400	-1.47241600	0.00184800
H	4.11053700	-2.54180800	0.00149900
C	3.13681700	-0.60235500	0.00026100
C	3.37134800	0.78085000	0.00096300
C	4.67850100	1.27159600	0.00318200
H	4.79439700	2.34650500	0.00365000
C	0.94521800	1.11897600	-0.00256600
H	0.09805100	1.78403100	-0.00355800
C	2.24959800	1.73918100	-0.00056800
C	0.76290300	-0.22543200	-0.00286700
N	1.83472100	-1.09420700	-0.00180700
O	2.42377400	2.94884800	-0.00029900
C	1.66846600	-2.54233500	-0.00275100
H	0.62069400	-2.80316100	-0.00489600
H	2.12844800	-2.97424900	0.88612000
H	2.13176000	-2.97335500	-0.89033800
C	-0.62306300	-0.82720200	-0.00393400
H	-0.72681900	-1.47214900	-0.88137800
H	-0.72764100	-1.47249200	0.87319100
C	-1.77766700	0.16868800	-0.00434900

H	-1.71262600	0.81474800	0.87350900
H	-1.71428100	0.81197700	-0.88439600
C	-3.12329700	-0.55272000	-0.00147500
H	-3.18870900	-1.20742500	-0.87618300
H	-3.18855400	-1.19961600	0.87905700
C	-4.30654200	0.41037900	-0.00573100
H	-4.24100800	1.06864200	0.86578400
H	-4.24609700	1.05457300	-0.88807200
C	-5.65212800	-0.30873500	0.00415500
H	-5.71632500	-0.97030000	-0.86561000
H	-5.71313800	-0.95118300	0.88837900
C	-6.84008100	0.64910900	-0.00421200
H	-6.77319500	1.31121800	0.86286900
H	-6.77984600	1.28762700	-0.88930700
C	-8.17726400	-0.08619300	0.01078000
H	-8.26469800	-0.70951800	0.90214000
H	-8.26989700	-0.73650200	-0.86052400
H	-9.01657000	0.60865200	0.00261500

3-[4-(4- methylhydroxy-2-butenyloxy)-phenyl]-2-propanol (**2**)

C	-1.16373800	0.17457300	0.81611900
H	-1.65379000	0.91735200	1.42990800
C	-1.82917900	-0.35946500	-0.28758700
C	-1.13692600	-1.27840400	-1.08166800
H	-1.62357200	-1.69843400	-1.95245000

C	0.15469700	-1.66712900	-0.77532000
H	0.69084700	-2.38384600	-1.38149200
C	0.79504600	-1.13694600	0.34437200
C	0.13203900	-0.20192400	1.13776300
H	0.61800000	0.24744700	1.98950800
O	2.05898600	-1.58561500	0.56614500
C	-3.20714700	0.00270500	-0.64521200
H	-3.51676800	-0.29573300	-1.64309300
C	-4.09505700	0.63104900	0.12420600
H	-3.85558300	0.91441200	1.14351600
C	-5.48565400	0.95386100	-0.32444200
H	-5.63838400	0.60122600	-1.34934000
H	-5.65374100	2.03038800	-0.30899600
O	-6.45769300	0.40980900	0.56140500
H	-6.28111800	-0.52982200	0.63290300
C	2.84263300	-0.95844700	1.58538800
H	3.74339700	-1.56755200	1.63068800
H	2.33188000	-1.05129500	2.54573700
C	3.16598400	0.48795600	1.29956500
H	3.00742500	1.18421600	2.11669700
C	3.60428500	0.96285000	0.13471000
C	3.81880800	0.09463500	-1.08179300
H	3.90402400	-0.95748800	-0.81463500
H	4.73464200	0.40403200	-1.58544100
C	3.83135700	2.42739000	-0.09851300
H	4.88228900	2.61654100	-0.32908200

H	3.54723900	3.02615600	0.76467400
H	3.24724800	2.74402400	-0.96394300
O	2.77398800	0.29717600	-2.02777100
H	1.95083300	0.03119600	-1.60892000

Table S3. PTP1B and α-glucosidase inhibitory activities of total methanol extracts and its fractions from *T. ruticarpum*

Compounds	PTP1B	α-glucosidase
	% Inhibition, μg/mL[a]	% Inhibition, μg/mL[a]
**MeOH extract**	53.2 ± 1.8	ND[b]
**EtOAc fraction**	67.7 ± 2.3	-
**H_2O fraction**	23.5 ± 0.6	-
TR1	-[b]	47.9 ± 1.3
TR2	-	57.5 ± 2.7
TR3	-	< 20
TR4	-	< 20
TR5	-	27.3 ± 2.4
TR6	-	43.6 ± 2.1
TR7	-	< 20
TR8	-	< 20
TR9	-	44.8 ± 3.2
TR10	-	52.6 ± 2.9

[a] Results are expressed as % inhibition with the final tested concentration of 30 μg/mL for the testing sample (n = 2).

[b] Data not determined.
